# Decreased YAP activity reduces proliferative ability in human induced pluripotent stem cell of duchenne muscular dystrophy derived cardiomyocytes

**DOI:** 10.1038/s41598-021-89603-8

**Published:** 2021-05-14

**Authors:** Hideki Yasutake, Jong-Kook Lee, Akihito Hashimoto, Kiyoshi Masuyama, Jun Li, Yuki Kuramoto, Shuichiro Higo, Shungo Hikoso, Kyoko Hidaka, Atsuhiko T. Naito, Shigeru Miyagawa, Yoshiki Sawa, Issei Komuro, Yasushi Sakata

**Affiliations:** 1 Department of Cardiovascular Medicine, Osaka University Graduate School of Medicine, 2-2 Yamadaoka, Suita, 565-0871 Japan; 2 Department of Cardiovascular Regenerative Medicine, Osaka University Graduate School of Medicine, 2-2 Yamadaoka, Suita, 565-0871 Japan; 3 Daihatsu Health Care Center, 2-1-1 Momozono, Ikeda, 563-0045 Japan; 4 Department of Medical Therapeutics for Heart Failure, Osaka University Graduate School of Medicine, 2-2 Yamadaoka, Suita, Osaka 565-0871 Japan; 5 Center for Fundamental Education, The University of Kitakyushu, 4-2-1 Kokura Minami-ku Kitagata, Kitakyushu, 802-8577 Japan; 6 Department of Physiology, Faculty of Medicine, Toho University, 5-21-16 Omori-nishi, Ohta-ku, Tokyo, 143-8540 Japan; 7 Department of Cardiovascular Surgery, Osaka University Graduate School of Medicine, 2-2 Yamadaoka, Suita, 565-0871 Japan; 8 Department of Cardiovascular Medicine, Graduate School of Medicine, The University of Tokyo, 7-3-1 Hongo, Bunkyo-ku, Tokyo, 113-8655 Japan

**Keywords:** Cardiology, Cardiovascular biology, Cardiovascular diseases, Cardiomyopathies, Heart failure

## Abstract

Duchenne muscular dystrophy (DMD) is characterized by progressive muscle degeneration accompanied by dilated cardiomyopathy. Recently, abnormality of yes-associated protein (YAP) has been reported as the pathogenesis of muscle degeneration of DMD; however YAP activity remains unclear in dystrophic heart of DMD. Herein, we investigated YAP activity using disease-specific induced pluripotent stem cell (iPSC) derived cardiomyocytes (CMs) in DMD. DMD-iPSCs were generated from DMD patient with exon 48–54 deletion in *DMD*, and genome-edited (Ed)-DMD-iPSCs with in-frame (Ed-DMD-iPSCs) were created using CRISPR/Cas9. Nuclear translocation of YAP [nuclear (N)/cytoplasmic (C) ratio] was significantly lower in DMD-iPSC-CMs than in Ed-DMD-iPSC-CMs. In addition, Ki67 expression, indicating proliferative ability, was significantly lower in DMD-iPSC-CMs than Ed-DMD-iPSC-CMs. Therefore, immunofluorescent staining showed that actin stress fibers associated with YAP activity by mechanotransduction were disorganized in DMD-iPSC-CMs. Lysophosphatidic acid (LPA), a known lipid mediator on induction of actin polymerization, significantly increased YAP activity and actin dynamics in DMD-iPSC-CMs using live cell imaging. These results suggested that altered YAP activity due to impaired actin dynamics reduced proliferative ability in DMD-iPSC-CMs. Hence, decreased YAP activity in dystrophic heart may contribute to DMD-cardiomyopathy pathogenesis.

## Introduction

Duchenne muscular dystrophy (DMD) is an X-linked genetic disease that affects 1 in 3500 males with degenerative muscular damage^1^. DMD is caused by defect in dystrophin protein encoded by *DMD*, a gene with 79 exons in the p21.1 region of the X chromosome. Both skeletal and cardiac muscles in DMD are fragile and most patients with DMD die of heart failure due to cardiomyopathy^1^. Defects in dystrophin excessively increase intracellular calcium concentration, reactive oxygen species production, and apoptosis with mitochondrial dysfunction^2–4^, however, the pathological mechanism underlying DMD is not clear.

Most mutations in *DMD* are deletions that occur in the “hot spot” between exons 45 and 55^5^. Currently, CRISPR/Cas9, a new genome editing technology, is being considered for DMD therapy. Dystrophin was repaired in animal models of DMD using CRISPR/Cas9^6^, and this technology has been used for elucidating the pathogenesis of various diseases. Disease-specific induced pluripotent stem cells (iPSCs) harboring mutations found in patients and isogenic controls of disease-specific iPSCs edited using CRISPR/Cas9 act as effective disease models^4,7,8^. The results obtained using disease-specific iPSCs established from patients with DMD (DMD-iPSCs) can complement the observations made in MDX mice, the animal model of DMD, as the phenotypes of MDX mice and human differ^1,9^. A previous study has demonstrated that dystrophin repaired using CRISPR/Cas9 improved the abnormal phenotype in DMD-iPSCs^10,11^. Thus, CRISPR/Cas9 and DMD-iPSCs are important tools for elucidating the pathogenesis of DMD.

Yes-associated protein (YAP) is a transcriptional cofactor that promotes cell proliferation, organ size during growth, and regeneration^12^. It has been reported that YAP promotes cardiomyocyte proliferation and regeneration via regulation of the Hippo pathway, which consists of mammalian Ste20-like (MST) or large tumor suppressor (LATS) kinase^12^. Hippo pathway that regulates YAP is expected as molecular target for heart failure^13^. A recent study showed that YAP activity is suppressed in muscle of patients with DMD, and that poor regeneration ability associated with decreased YAP activity causes muscular damage in dystrophic skeletal muscles^14^. However, YAP activity has not been reported in the dystrophic heart of patients with DMD, and whether DMD cardiomyopathy is related to YAP activity is not known. Activated YAP translocates from the cytoplasm to the nuclei and promotes gene expression associated with cell proliferation and anti-apoptosis^12,15^. YAP exhibits mechanosensitive activity by sensing the features of the surrounding microenvironment, such as cell density and substrate stiffness^16,17^. YAP activity increases due to mechanotransduction, which converts physical stimulus to electrochemical stimulus^18^. Mechanotransduction is regulated by the actin filament, an important cytoskeletal stress fibers organized via the dynamics between F actin and G actin^19,20^. YAP activity changes due to actin dynamics, which is modulated by the G protein-coupled receptor (GPCR) signaling pathway^21^. Lysophosphatidic acid (LPA), a phospholipid mediator that acts through GPCR, was reported to promotes actin dynamics via Rho-Rock kinase and increase YAP activity and cell proliferation^22,23^.

Dystrophin is a cytoskeletal component anchoring actin stress fibers to the dystrophin-glycoprotein complex (DGC). We hypothesized that defects in dystrophin in patients with DMD may result in dysfunction of actin stress fibers and inhibit YAP activity. We investigated YAP activity and actin dynamics using disease-specific iPSC-derived cardiomyocytes generated from patients with DMD (DMD-iPSC-CMs) compared to iPSC-CMs generated from a healthy human control (Con-iPSC-CMs) and genome-edited (Ed)-DMD-iPSC-CMs repaired using CRISPR/Cas9 (Ed-DMD-iPSC-CMs). Moreover, we analyzed actin dynamics by treatment of LPA using live cell imaging in DMD-iPSC-CMs.

## Results

### Genome editing in DMD-iPSCs using CRISPR/Cas9

**Figure 1:**
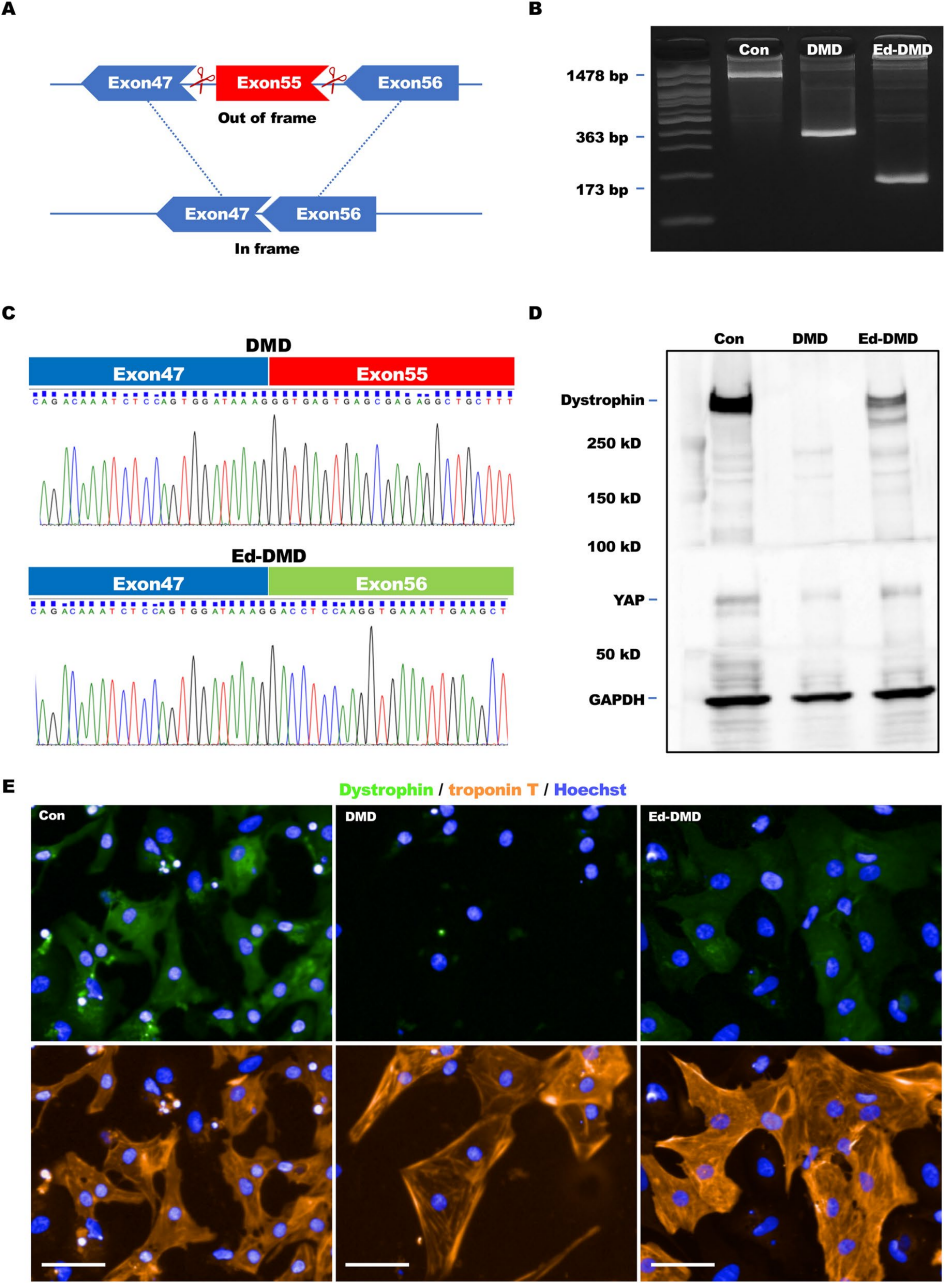
Repair of dystrophin in DMD-iPSC-CMs using CRISPR/Cas9. (**A**) Strategy of genome editing using CRISPR/Cas9. Exon 55 in DMD-iPSCs was deleted using each sgRNAs designed upstream and downstream of exon 55 to modify from “out of frame” to “in frame.” (**B**) Exon size from exon 47 to exon 56 in Con-iPSCs, DMD-iPSCs, and Ed-DMD-iPSCs determined using PCR with complementary DNA (cDNA) as the template. (**C**) Sanger sequencing of DMD-iPSCs (Δexon 48–54) and Ed-DMD-iPSCs (Δexon 48–55). (**D**,**E**) Expression of dystrophin in Con-iPSC-CMs, DMD-iPSC-CMs, and Ed-DMD-iPSC-CMs determined using western blotting and immunofluorescence staining (scale bar 50 µm).

Disease-specific iPSCs, previously generated from DMD patient with exon 48–54 deletion who had heart failure^24^, were used in this study. Additionally, 201B7 cells (RIKEN BRC Cell Bank, Tsukuba, Japan) generated from a healthy human, were used as Con-iPSCs. We confirmed the exon deletion area of *DMD* in DMD-iPSCs. Genotyping of the genomic DNA of iPSCs showed that compared to Con-iPSCs, DMD-iPSCs harbored deletion from exons 48 to 54 (Figure I in Data Supplement). This was consistent with the clinical diagnosis of the patient. Next, we attempted to edit the genome of DMD-iPSCs. Each sgRNA on introns both upstream and downstream of exon 55 was designed to modify “out of frame” mutation to “in frame” using exon skipping via double site genome editing in this study (Fig. 1A). pX459, encoding each sgRNA, was co-transfected into DMD-iPSCs via electroporation. We selected certain co-transfected colonies and performed genotyping to acquire Ed-DMD-iPSCs. PCR with complementary DNA (cDNA) as the template, with forward primer on exon 47 and reverse primer on exon 56 showed that the exon sizes of Con-iPSCs, DMD-iPSCs, and Ed-DMD-iPSCs were 1478 bp, 363 bp, and 173 bp, respectively (Fig. 1B). This showed that exon 55 of 190 bp was resected in Ed-DMD-iPSCs. Sanger sequencing showed that exons 47 and 56 were connected in Ed-DMD-iPSCs (Fig. 1C). Thus, we acquired Ed-DMD-iPSCs with the expected genome editing.

### Cardiomyocyte differentiation from iPSCs

We generated iPSC-CMs according to the cardiomyocyte differentiation protocol. The expression of dystrophin in Con-iPSC-CMs was confirmed at each time point of cardiac differentiation. Troponin T was used for labeling the cardiomyocytes in Con-iPSC-CMs. The percentages of dystrophin-positive cells in troponin T-positive cells were 40%, 69%, and 91% after 10, 20, and 30 days of differentiation (Figure IIA–C in Data Supplement). The expression of dystrophin was low in 10-days-old iPSC-CMs, though troponin-T was expressed. This showed that 10-days-old iPSC-CMs were inappropriate for studying the function of dystrophin and iPSC-CMs > 30 days after differentiation should be used. Next, we confirmed the expression of dystrophin in DMD-iPSC-CMs and Ed-DMD-iPSC-CMs 30 days after differentiation. Dystrophin expression was defective in DMD-iPSC-CMs, while full length dystrophin was expressed in Con-iPSC-CMs and short length dystrophin with deletion of exon 48–55 was expressed in Ed-DMD-iPSC-CMs, as observed using western blotting (Fig. 1D) and immunofluorescence staining (Fig. 1E). Short length dystrophin was repaired through exon skipping, which modified *DMD* from “out of frame” to “in frame”. Therefore, we used iPSC-CMs 30 days post differentiation (iPSC-CMs 30 days) in all the subsequent experiments.

### Analysis of YAP localization in DMD-iPSC-CMs

**Figure 2:**
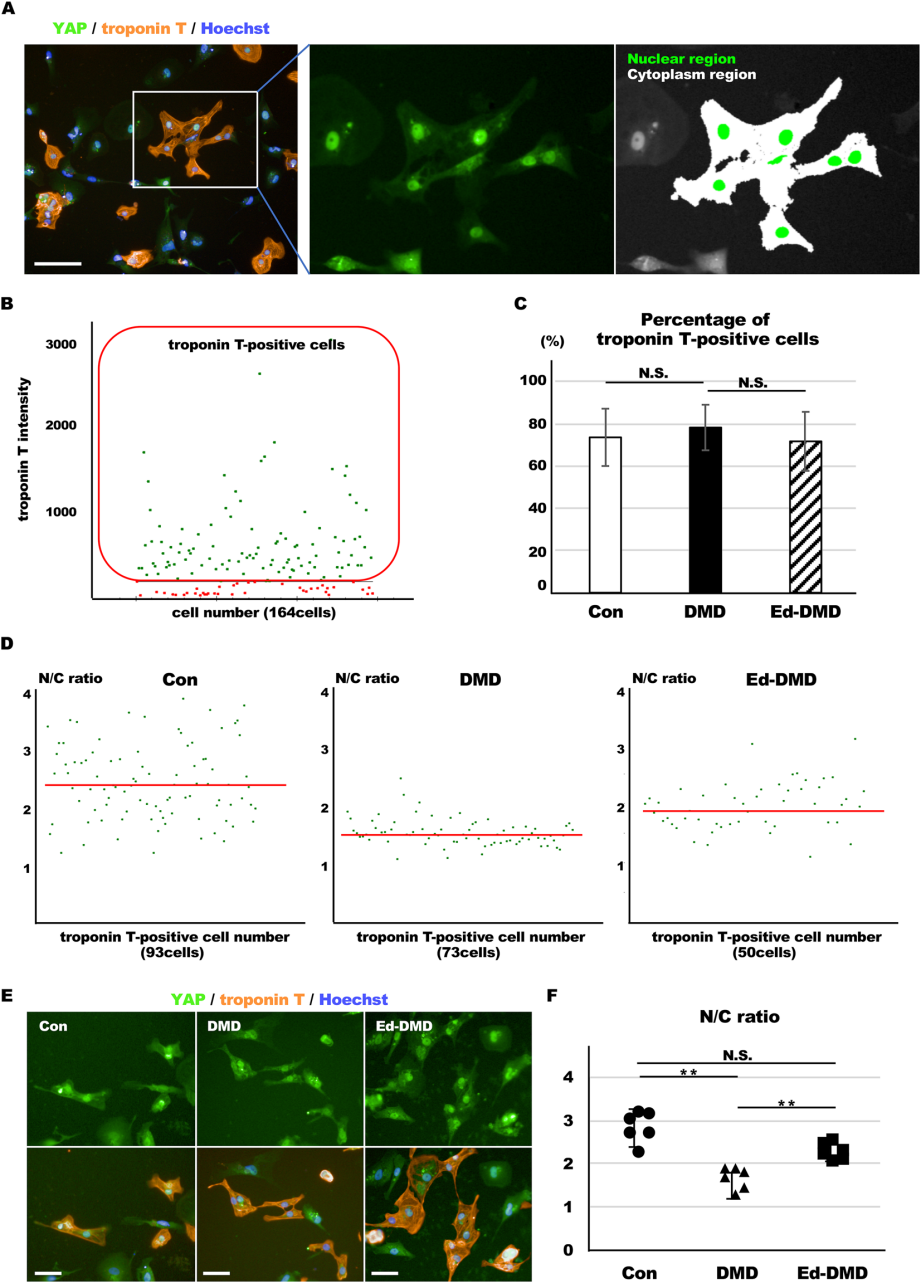
Analysis of YAP activity in DMD-iPSC-CMs. (**A**) YAP localization was analyzed using immunofluorescence staining. The N/C ratio is the intensity of the YAP nuclear region divided by the cytoplasmic intensity in iPSC-CMs labeled using troponin T (scale bar 100 µm). (**B**) Scatter plots of one field in Con-iPSC-CMs labeled using troponin T. Red frame indicates troponin T-positive cells. (**C**) Percentage of troponin T-positive cells was evaluated in Con-iPSC-CMs, DMD-iPSC-CMs, and Ed-DMD-iPSC-CMs (n = 6 sessions, mean analyzed cell number = 2971 ± 1965, 1546 ± 953, and 3566 ± 2889 cells, respectively, mean ± SD). (**D**) Scatter plots of one field in Con-iPSC-CMs, DMD-iPSC-CMs and Ed-DMD-iPSC-CMs. Red bars indicate mean N/C ratio in troponin T-positive cells. (**E**) YAP localization demonstrated using immunofluorescent staining in Con-iPSC-CMs, DMD-iPSC-CMs, and Ed-DMD-iPSC-CMs (scale bar 50 µm). (**F**) N/C ratio in troponin T-positive cells was evaluated in Con-iPSC-CMs, DMD-iPSC-CMs and Ed-DMD-iPSC-CMs (n = 6 sessions, mean analyzed troponin T-positive cell number = 985 ± 576, 805 ± 716, and 744 ± 845 cells, respectively, mean ± SD, **P < 0.01, *N.S.* not significantly different).

We confirmed YAP localization in iPSC-CMs. Twenty-four hours after seeding iPSC-CMs on the imaging plate, YAP was detected using immunofluorescence staining and analyzed using the high content imaging system. YAP mostly accumulated in the nuclei, and not the cytoplasm, of Con-iPSC-CMs (Fig. 2A). For detailed analysis of YAP localization, we estimated the nuclear/cytoplasmic (N/C) ratio from the nuclear region YAP intensity/cytoplasm region YAP intensity in iPSC-CMs labeled using troponin T (Fig. 2A). The imaging analysis was performed in a number of iPSC-CMs of the 59 fields; the size of one field was 500 µm × 650 µm. We confirmed the percentages of troponin T-positive cells in iPSC-CMs 30 days. The percentages of troponin T-positive cells were 73%, 78%, and 71% in Con-iPSC-CMs, DMD-iPSC-CMs, and Ed-DMD-iPSC-CMs 30 days, respectively. There was no significant difference among these iPSC-CMs (Fig. 2B,C). We estimated the changing of N/C ratio by the microenvironment in terms of cell density and substrate stiffness. High cell density (1 × 10^5^ cells in 96-well plate) resulted in lower N/C ratio than low cell density (1 × 10^4^ cells in 96-well plate) in troponin T-positive cells in Con-iPSC-CMs (2.39 ± 0.8 vs. 1.53 ± 0.09, *P* < 0.05; Figure IIIA–C in Data Supplement). In substrate stiffness which ranged from 0.5 to 32 kPa using biocompatible silicone, hard substrate (32 kPa) showed higher N/C ratio than soft substrate (0.5 kPa) in Con-iPSC-CMs (2.17 ± 0.15 vs 3.13 ± 0.60, *P* < 0.05; Figure IVA–C in Data Supplement). These results indicated changing of YAP activity due to contact inhibition with cell density and mechanotransduction through substrate stiffness. Hence, we determined the N/C ratio in Con-iPSC-CMs, DMD-iPSC-CMs, and Ed-DMD-iPSC-CMs to assess YAP activity. The N/C ratio in DMD-iPSC-CMs was lower than that of Con-iPSC-CMs and Ed-DMD-iPSC-CMs (2.88 ± 0.34 vs. 1.67 ± 0.24 vs. 2.31 ± 0.19, *P* < 0.01; Fig. 2D–F). These results showed that YAP activity decreased in DMD-iPSC-CMs and improved in Ed-DMD-iPSC-CMs.

### Proliferative ability of DMD-iPSC-CMs

**Figure 3:**
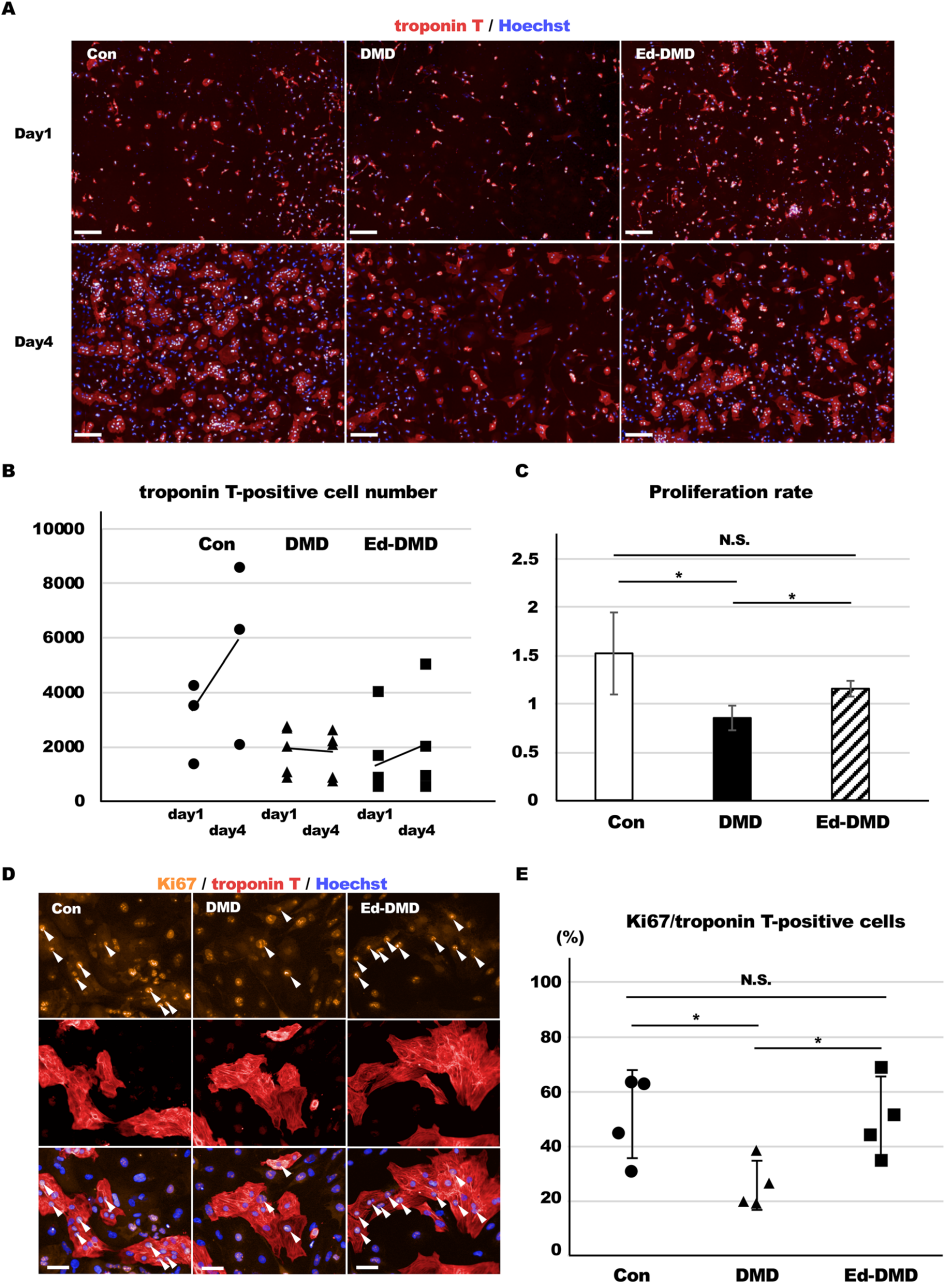
Proliferative ability of DMD-iPSC-CMs. (**A**) Troponin T-positive cells was identified using immunofluorescence staining in Con-iPSC-CMs, DMD-iPSC-CMs, and Ed-DMD-iPSC-CMs, on days 1 and 4 post seeding (scale bar 200 µm). (**B**) Number of troponin T-positive cells according to the time course from day 1 to day 4 (n = 4 sessions). (**C**) Proliferation rate was evaluated in Con-iPSC-CMs, DMD-iPSC-CMs, and Ed-DMD-iPSC-CMs (n = 4 sessions, mean ± SD, *P < 0.05,* N.S*. not significantly different). (**D**) Ki67 expression was detected using immunofluorescence staining in Con-iPSC-CMs, DMD-iPSC-CMs, and Ed-DMD-iPSC-CMs (scale bar 50 µm). Arrowheads indicate Ki67-positive cells in troponin T-positive cells. (**E**) Percentage of Ki67-positive cells in troponin T-positive cells was evaluated in Con-iPSC-CMs, DMD-iPSC-CMs, and Ed-DMD-iPSC-CMs (n = 4 sessions, mean analyzed troponin T-positive cell number = 1974 ± 651, 1560 ± 971, and 3512 ± 3547 cells, respectively, mean ± SD, *P < 0.05, *N.S*. not significantly different).

We assessed the cardiomyocyte proliferation rate of the iPSC-CMs. We counted the number of troponin T-positive cells, 1 day after seeding the iPSC-CMs (2 × 10^4^ cells in 96-well plate), using immunofluorescence staining to determine the baseline number. The iPSC-CMs were cultured for 4 days and the number of troponin T-positive cells was counted using immunofluorescence staining in the same session of cardiac differentiation. The proliferation rate of iPSC-CMs was evaluated as the number of troponin T-positive cells in the baseline/the number of troponin T-positive cells after 4 days. DMD-iPSC-CMs exhibited significantly lower proliferation rate than Con-iPSC-CMs and Ed-DMD-iPSC-CMs (1.52 ± 0.42 vs. 0.85 ± 0.12 and 1.16 ± 0.08, *P* < 0.05; Fig. 3A–C). We assessed the proliferative ability of these iPSC-CMs using Ki67 expression as a cell cycle marker. Ki67 expression was evaluated in troponin T-positive cells in Con-iPSC-CMs on imaging plates 1 day and 4 days from seeding to confirm the phase of the cell cycle. Ki67 expression in troponin T-positive cells was negligible in Con-iPSC-CMs after 1 day, whereas Ki67 expression in troponin T-positive cells significantly increased after 4 days (7 ± 3% vs. 33 ± 4%, *P* < 0.001, Figure VA–C in Data Supplement). This showed that Con-iPSC-CMs were in the resting phase of the cell cycle for at least 1 day after seeding; however, proliferation of Con-iPSC-CMs happened from day 1 to day 4. Therefore, we assessed Ki67 expression in troponin T-positive cells in these iPSC-CMs after 4 days to evaluate their proliferative ability. DMD-iPSC-CMs showed significantly lower Ki67-positive cells in troponin T-positive cells than Con-iPSC-CMs and Ed-DMD-iPSC-CMs (51 ± 16% vs. 26 ± 9% vs. 50 ± 14%, *P* < 0.05, Fig. 3D,E). These results showed that DMD-iPSC-CMs possess poor proliferative ability, which might be because of decreased YAP activity.

### Cell morphology and actin filament status in DMD-iPSC-CMs

**Figure 4:**
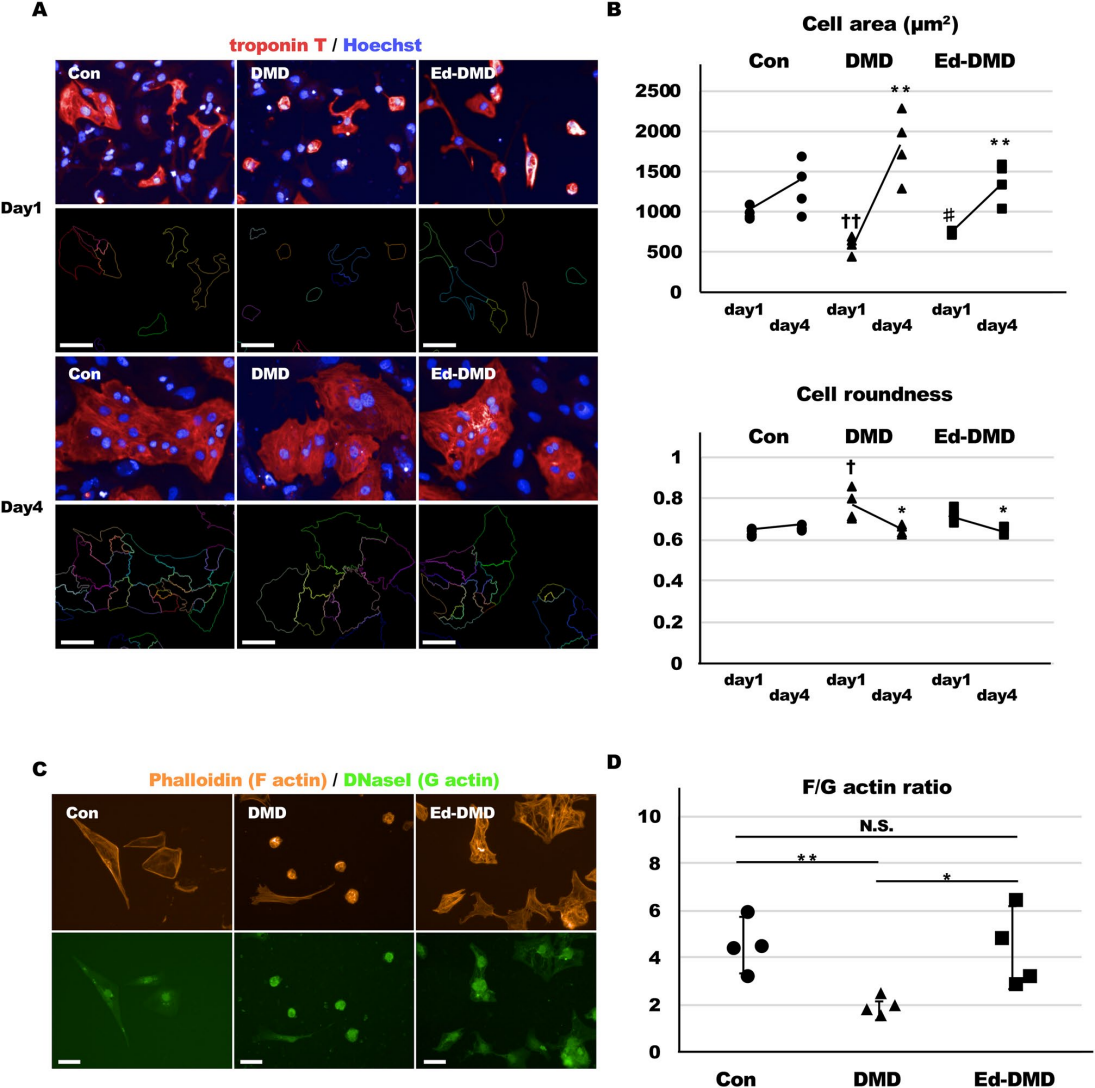
Actin stress fibers and cell morphology in DMD-iPSC-CMs. (**A**) Cell morphology was analyzed using immunofluorescence staining. Trace of cell shape in Con-iPSC-CMs, DMD-iPSC-CMs, and Ed-DMD-iPSC-CMs labeled using troponin T on days 1 and 4 post seeding (scale bar 50 µm). (**B**) Cell area and roundness of troponin T-positive cells according to the time course from days 1 to 4 (n = 4 sessions, mean analyzed troponin T-positive cell number = 68 ± 21, 27 ± 9, and 63 ± 24 cells, respectively, mean ± SD, *P < 0.05, **P < 0.01 day 1 vs. day 4, ^†^*P* < 0.05, ^††^*P* < 0.01 Con-iPSC-CMs vs. DMD-iPSC-CMs, ^#^*P* < 0.05 DMD-iPSC-CMs vs. Ed-DMD-iPSC-CMs). (**C**) F actin and G actin in Con-iPSC-CMs, DMD-iPSC-CMs, and Ed-DMD-iPSC-CMs, day1 from seeding were visualized using immunofluorescence staining. F actin and G actin were labeled using phalloidin and DNase I, respectively (scale bar 50 µm). (**D**) F/G actin ratio in troponin T-positive cells was evaluated by dividing the intensity of phalloidin by the intensity of DNase I in the cytoplasmic region of Con-iPSC-CMs, DMD-iPSC-CMs, and Ed-DMD-iPSC-CMs, day1 post seeding (n = 4 sessions, mean analyzed troponin T-positive cell number = 649 ± 80, 750 ± 253, and 730 ± 261 cells, respectively, mean ± SD, *P < 0.05, *N.S*. not significantly different).

We assessed the actin state to determine whether the actin filament was associated with altered YAP activity in DMD-iPSC-CMs. We recorded the cell morphology in troponin T-positive cells in the iPSC-CMs on day 1 and day 4 after seeding, using immunofluorescence staining. Troponin T-positive cells in DMD-iPSC-CMs were smaller and rounder on day 1 than those in Con-iPSC-CMs and Ed-DMD-iPSC-CMs (*P* < 0.05, Fig. 4A,B). Morphology of troponin T-positive cells in DMD-iPSC-CMs drastically changed between day 1 and day 4, compared to those in Con-iPSC-CMs and Ed-DMD-iPSC-CMs (*P* < 0.01, Fig. 4A,B). However, iPSC-CMs exhibited an expanded cell shape on day 4, while the cell morphology parameters in troponin T-positive cells in DMD-iPSC-CMs were not significantly different from those in Con-iPSC-CMs and Ed-DMD-iPSC-CMs (Fig. 4A,B). Con-iPSC-CMs and Ed-DMD-iPSC-CMs were multinucleated, while DMD-iPSC-CMs had lower number of nuclei. These results indicated that the change in the morphology between the cells on day 1 and day 4 was because of the varying proliferative ability in the iPSC-CMs. In addition, we evaluated the actin state in troponin T-positive cells in iPSC-CMs on day 1. Immunofluorescence staining showed that the structure of F actin in actin stress fibers in DMD-iPSC-CMs was disrupted compared to that in Con-iPSC-CMs and Ed-DMD-iPSC-CMs after 1 day (Fig. 4C). The ratio of F actin intensity to G actin intensity (F/G actin) decreased significantly in DMD-iPSC-CMs (4.58 ± 1.09 vs. 1.96 ± 0.39 vs 4.42 ± 1.64, *P* < 0.05, Fig. 4D). These results indicated that impairment of actin stress fibers might affect cell morphology and decrease YAP activity in DMD-iPSC-CMs by reducing mechanotransduction.

### Effect of LPA for DMD-iPSC-CMs

**Figure 5:**
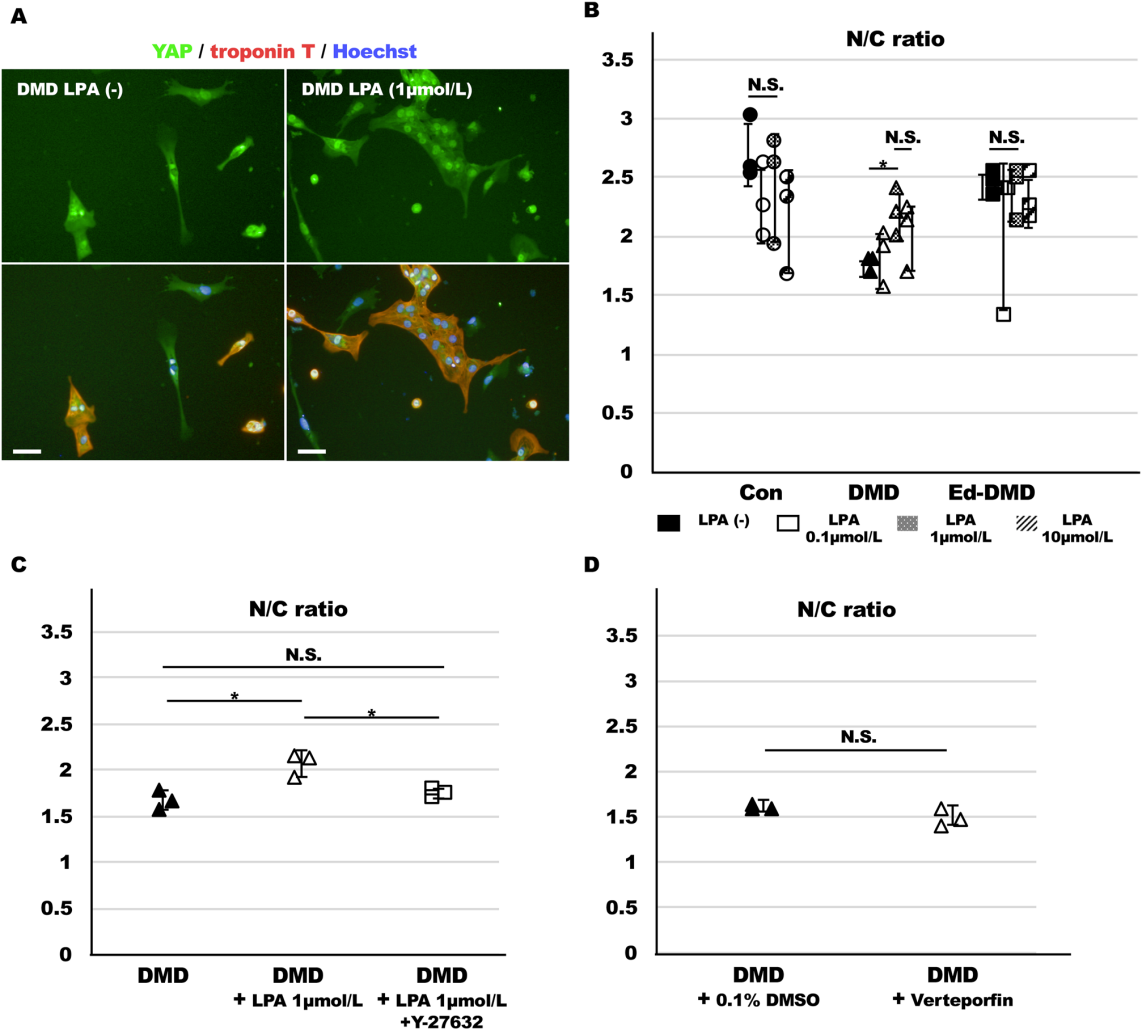
Effect of LPA on YAP activity in DMD-iPSC-CMs. (**A**) YAP localization was determined in DMD-iPSC-CMs, not treated, and treated with LPA (scale bar 50 µm). (**B**) N/C ratio in troponin T-positive cells was evaluated at various concentrations of LPA in Con-iPSC-CMs, DMD-iPSC-CMs, and Ed-DMD-iPSC-CMs (n = 3 sessions, mean analyzed troponin T-positive cell number = 569 ± 177, 320 ± 175, and 271 ± 123 cells, respectively, mean ± SD, *P < 0.05, *N.S*. not significantly different). (**C**) N/C ratio in troponin T-positive cells was evaluated for not treated, treated with 1 µmol/L LPA, and treated with 1 µmol/L LPA and 10 µmol/L Y-27632 in DMD-iPSC-CMs (n = 3 sessions, mean analyzed troponin T-positive cell number = 563 ± 77, 302 ± 179, and 235 ± 133 cells, respectively, mean ± SD, *P < 0.05, *N.S*., not significantly different). (**D**) N/C ratio in troponin T-positive cells was evaluated for treatments with 0.1% DMSO and 10 µmol/L verteporfin in DMD-iPSC-CMs (n = 3 sessions, mean analyzed troponin T-positive cell number = 824 ± 143 and 852 ± 618 cells, respectively, mean ± SD, *N.S*., not significantly different).

We investigated whether LPA (Sigma-Aldrich) that promotes actin dynamics via Rho GTPase improved YAP activity and proliferative ability of DMD-iPSC-CMs. We treated iPSC-CMs with various concentrations of LPA (0, 0.1, 1. 10 µmol/L). iPSC-CMs on the imaging plate were treated with LPA for 2 days after seeding and the N/C ratio in troponin T-positive cells in iPSC-CMs was determined. The N/C ratio in DMD-iPSC-CMs increased after 1 µmol/L LPA treatment, while none of the LPA concentrations increased N/C ratio in Con-iPSC-CMs and Ed-DMD-iPSC-CMs. Interestingly, N/C ratio in DMD-iPSC-CMs increased after treatment with 1 µmol/L LPA (without LPA vs. 1 µmol/L LPA in DMD-iPSC-CMs; 1.76 ± 0.06 vs. 2.20 ± 0.19; *P* < 0.05; Fig. 5A,B), but plateaued after treatment with 10 µmol/L LPA. Improvement of N/C ratio by 1 µmol/L LPA treatment was reversed by 10 µmol/L Y-27632 (Wako), a ROCK inhibitor, in DMD-iPSC-CMs (*P* < 0.05, Fig. 5C). These results indicated that LPA partially improved YAP activity in DMD-iPSC-CMs, but not in Con-iPSC-CMs and ED-DMD-iPSC-CMs. In addition, we investigated the effect of verteporfin (Cayman Chemical), a YAP inhibitor associated with disruption of YAP-TEAD interaction. N/C ratio did not change significantly with 10 µmol/L verteporfin in DMD-iPSC-CMs (Fig. 5D). Verteporfin did not affect YAP translocation from the cytoplasm to the nuclei, possibly because of direct YAP inhibitor-independent mechanotransduction.

**Figure 6:**
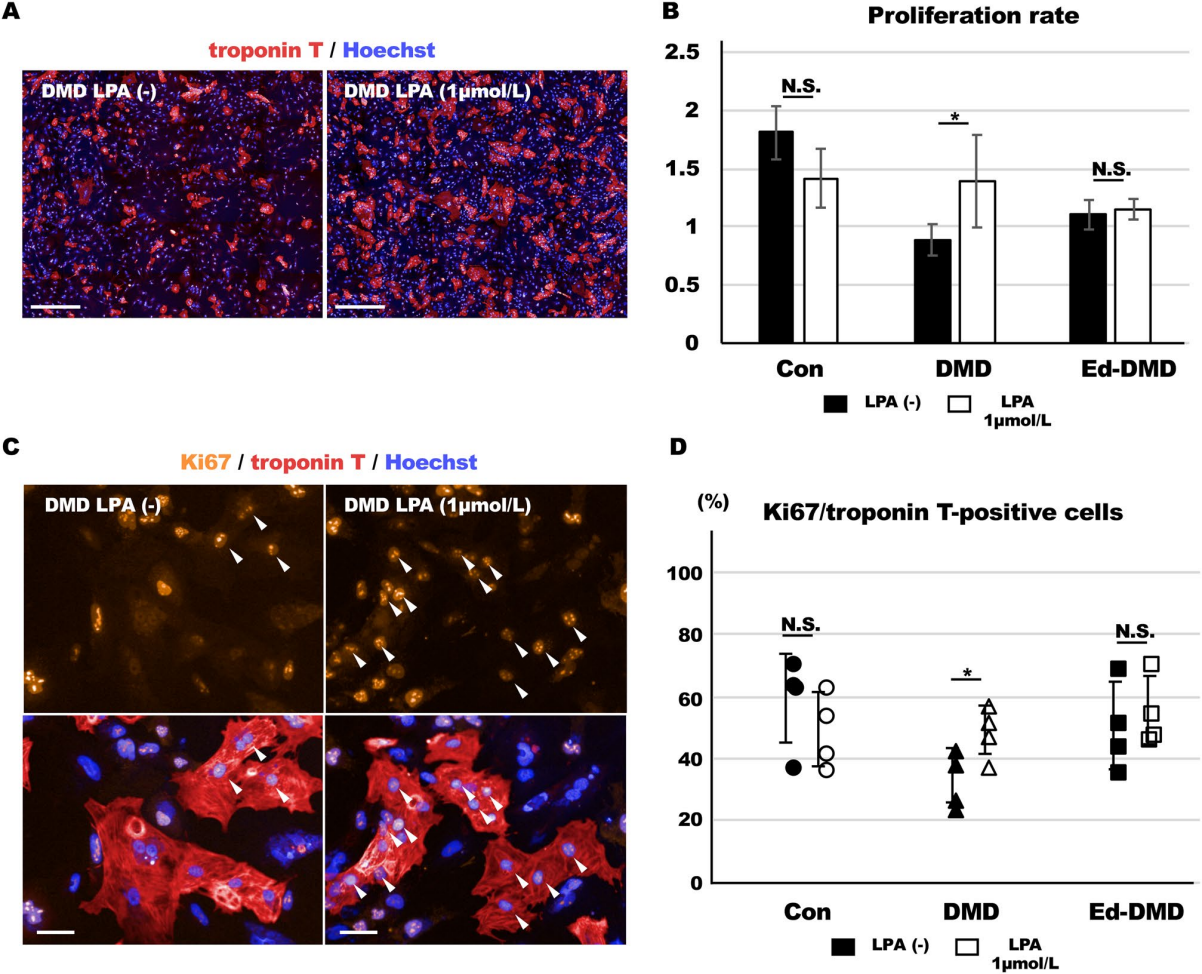
Proliferative ability of LPA-treated DMD-iPSC-CMs. (**A**) Troponin T-positive cells was identified using immunofluorescence staining in DMD-iPSC-CMs, not treated and treated with LPA (scale bar 500 µm). (**B**) Proliferation rate was evaluated in Con-iPSC-CMs, DMD-iPSC-CMs, and Ed-DMD-iPSC-CMs (n = 4 sessions, mean ± SD, *P < 0.05, *N.S*. not significantly different). (**C**) Ki67 expression was assessed using immunofluorescence staining in DMD-iPSC-CMs, not treated and treated with LPA. Arrowheads indicate Ki67-positive cells in troponin T-positive cells (scale bar 50 µm). (**D**) Ratio of Ki67-positive cells in troponin T-positive cells was evaluated in Con-iPSC-CMs, DMD-iPSC-CMs, and Ed-DMD-iPSC-CMs, not treated and treated with LPA (n = 4 sessions, mean analyzed troponin T-positive cell number = 2924 ± 1155, 2454 ± 339, and 2666 ± 2110 cells, respectively, mean ± SD, *P < 0.05, *N.S.* not significantly different).

We also confirmed whether LPA improved the proliferative ability of DMD-iPSC-CMs. The proliferation rates of these iPSC-CMs were evaluated by treating them with 1 µmol/L LPA for 2 days. LPA improved the proliferation rate of DMD-iPSC-CMs (1 µmol/L LPA treatment vs. control without LPA, *P* < 0.05, Fig. 6A,B). However, LPA did not significantly increase the proliferation rate of Con-iPSC-CMs and Ed-DMD-iPSC-CMs. In addition, the Ki67 expression was assessed in troponin T-positive cells in these iPSC-CMs in response to treatment with 1 µmol/L LPA for 2 days. Ki67 expression was significantly higher in LPA-treated DMD-iPSC-CMs than in untreated DMD-iPSC-CMs (without LPA vs. 1 µmol/L LPA, 33 ± 9% vs. 48 ± 8%, *P* < 0.05, Fig. 6C,D). Ki67 expression in Con-iPSC-CMs and Ed-DMD-iPSC-CMs did not increase after LPA treatment. These results indicated that LPA improved YAP activity and proliferative ability of DMD-iPSC-CMs.

### Actin dynamics in DMD-iPSC-CMs using live cell imaging

**Figure 7:**
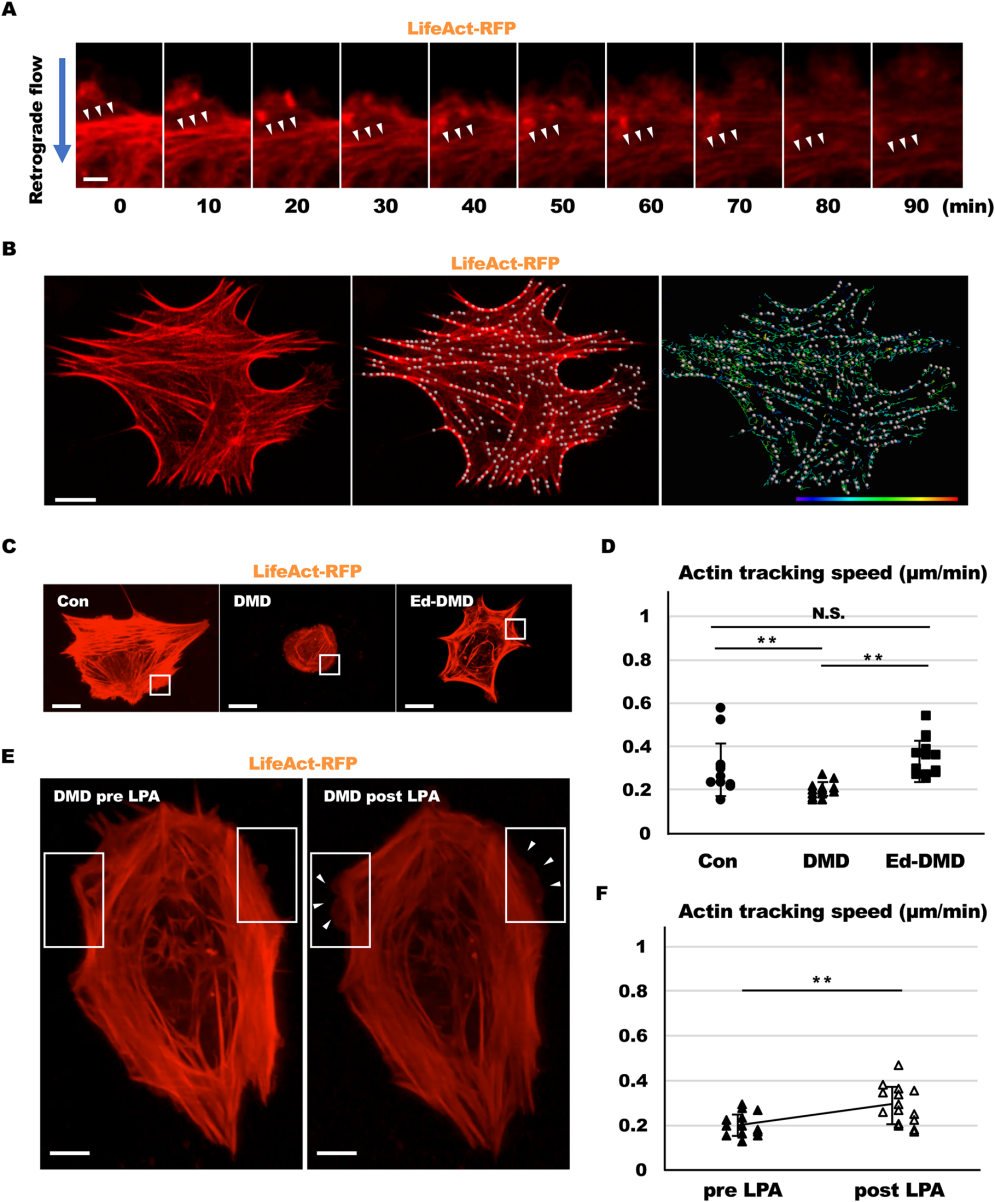
Actin dynamics in DMD-iPSC-CMs using live cell imaging. (**A**) Actin retrograde flow with organization of actin stress fibers was demonstrated using time lapse series of a confocal laser scanning microscope. Blue arrow shows centripetal direction of actin retrograde flow. White arrowheads indicate actin stress fibers that move with actin retrograde flow (scale bar 2 µm). (**B**) Actin tracking speed of actin filament was analyzed using live cell imaging to estimate actin dynamics (scale bar 10 µm, color bar: blue and red indicate range from 0 to 0.72 µm/min). (**C**) Actin dynamics in Con-iPSC-CMs, DMD-iPSC-CMs and Ed-DMD-iPSC-CMs was demonstrated using live cell imaging (scale bar 20 µm) (Movies 4, 5, 6). White square frames show the field of cell edge where actin tracking speed was estimated. (**D**) Actin tracking speed was estimated at the cell edge of Con-iPSC-CMs, DMD-iPSC-CMs and Ed-DMD-iPSC-CMs (n = 5 sessions, 13 cells, mean ± SD, **P < 0.01, *N.S.* not significantly different). (**E**) Actin dynamics in DMD-iPSC-CMs pre-treated and post-treated with LPA was demonstrated using live cell imaging (scale bar 10 µm) (Movies 7, 8). White square frames show the field of cell edge where actin tracking speed was estimated. Arrowheads indicate activated actin dynamics at the cell edge of DMD-iPSC-CMs. (**F**) Actin tracking speed was estimated at the cell edge of DMD-iPSC-CMs pre-treated and post-treated with LPA (n = 7 session, 16 cells, mean ± SD, **P < 0.01).

We investigated organization of actin stress fibers via actin dynamics in iPSC-CMs using live cell imaging. pCAG–LifeAct–TagRFP and AAV2 encoding CMV-EGFP-TNNT2 were co-transfected in iPSC-CMs. EGFP expression merged troponin T immunofluorescent signals (Figure VIA in Data Supplement), and the EGFP was detected in the sarcomere structure in iPSC-CMs (Figure VIB in Data Supplement). Actin dynamics was assessed by observation of actin retrograde flow that are driven by myosin II filament in EGFP-positive cell 24 h after seeding on imaging plates in a time lapse series. The imaging showed actin retrograde flow with organization of actin stress fibers in iPSC-CMs (Fig. 7A). Actin dynamics with actin retrograde flow was activated during cell division in the non-cardiomyocytes of iPSCs (Movie 1 and Figure VII in Data Supplement). Actin dynamics with actin retrograde flow was quantified by estimating the tracking speed of actin filament (Fig. 7B). We measured the velocity of actin retrograde flow at the cell edge and in the cell body of iPSC-CMs, using actin tracking speed. Actin tracking speed was higher at the cell edge than in the cell body (cell edge vs. cell body, *P* < 0.01; Movie 2 and Figure VIIIA,B in Data Supplement) of iPSC-CMs. These results indicated that actin dynamics with actin retrograde flow was activated at the cell edge, and not in the cell body. In addition, actin retrograde flow was slower in cardiomyocytes compared to that in the non-cardiomyocytes of Con-iPSCs (cardiomyocytes vs. non-cardiomyocytes, *P* < 0.01; Movie 3 and Figure IXA,B in Data Supplement). These results were consistent with that of a previous report showing that activity of actin dynamics is reduced in cardiomyocytes than in non-cardiomyocytes^25^. Hence, we assessed actin tracking speed at the cell edge in Con-iPSC-CMs, DMD-iPSC-CMs and Ed-DMD-iPSC-CMs. We observed that actin tracking speed in DMD-PSC-CMs was lower than that in Con-iPSC-CMs and Ed-DMD-iPSC-CMs (0.29 ± 0.12 µm/min vs. 0.19 ± 0.03 µm/min and 0.34 ± 0.09 µm/min, *P* < 0.01; Fig. 7C,D, Movies 4, 5, 6). These results indicated that actin dynamics with actin retrograde flow was impaired in DMD-iPSC-CMs. Next, we analyzed whether LPA promoted activity of actin dynamics in DMD-iPSC-CMs. We measured actin tracking speed in DMD-iPSC-CMs pre- and post-treated with 1 µmol/L LPA. Actin tracking speed increased in post LPA-treated DMD-iPSC-CMs compared with pre LPA-treated DMD-iPSC-CMs (pre LPA vs post LPA, *P* < 0.01; Fig. 7E,F, Movies 7, 8). This showed that LPA improved activity of actin dynamics in DMD-iPSC-CMs.

## Discussion

DMD causes degenerative muscular disorder; however, the molecular mechanism in pathogenesis of DMD remains unclear. This study aimed to elucidate the molecular mechanism of progressive DMD cardiomyopathy using disease-specific human iPSC-CMs. Furthermore, DMD-iPSC-CMs showed decreased YAP activity with impaired actin dynamics and CRISPR/Cas9-mediated repair of dystrophin improved YAP activity and actin dynamics in DMD-iPSC-CMs.

Recently, abnormal regeneration was speculated as a possible mechanism leading to the DMD phenotype. YAP is a transcriptional cofactor that promotes the expression of proliferation- and regeneration-related genes regulated by the Hippo pathway, which senses various modules such as tight junctions or adherens junctions^26,27^. Decreased YAP activity have already been described in skeletal muscle models of DMD, where regeneration is known to be highly critical^28^. Human samples of patients with DMD also showed decreased YAP activity and impaired regeneration of the skeletal muscle^14^. Thus, impaired regeneration of skeletal muscle in DMD is one of the causes of dystrophic muscular disease progression in DMD. In contrast, whether impaired regeneration and YAP activity are associated with dystrophic heart in DMD is not clear. Generally, adult cardiomyocytes possess limited regenerative ability, although neonatal cardiomyocytes show higher proliferative ability than adult cardiomyocytes. It is controversial whether this limited regeneration ability of cardiomyocytes also possibly contributes to the pathogenesis of cardiac dysfunction. YAP activity decreased in the cardiac tissue of patients who were diagnosed with ischemic heart disease and non-ischemic heart disease^13^. Micro RNA 302–367, a non-coding RNA that inhibited multiple components of the Hippo pathway such as MST or LATS, promotes cardiac regeneration^29^. Thus, the Hippo pathway, which regulates YAP activity, is a prospective therapeutic target for heart failure. On the other hand, the DGC composed of dystrophin interacts with phosphorylated YAP in cardiomyocytes^30^, and agrin, required for neuromuscular organization, interacted with DGC to promotes YAP activity^31^. The association of dystrophin and regulation of YAP activity in the dystrophic heart is reported in some previous studies.

YAP possesses mechano-sensitivity, which is regulated by actin stress fibers^18^. Actin stress fibers are important cytoskeletal components regulating cell morphology, migration, or mitosis, and actin remodeling is essential for heart regeneration^32^. Impairment of actin stress fibers by cytochalasin D decreases YAP activity, and changes in cell morphology due to actin stress fibers strongly correlates with YAP activity^19^. Dystrophin is a cytoskeletal protein anchoring DGC and actin stress fibers. We hypothesized that defect in dystrophin is followed by impairment of actin stress fibers and heart regeneration. Actin stress fibers are organized via actin dynamics with actin retrograde flow driven by myosin II filament. Quantitative evaluation of actin retrograde flow is useful as an indicator of actin dynamics activity^33,34^. A previous report has shown that actin retrograde flow is slower in cardiomyocytes than in non-cardiomyocytes^25^. A cardiomyocyte is composed of sarcomeres with actin and myosin filament, which may result in reduced activity of actin dynamics compared to non-cardiomyocytes. In this study, defect in dystrophin disorganized actin stress fibers and impaired actin dynamics with actin retrograde flow, and decreased YAP activity in DMD-iPSC-CMs compared to Con-iPSC-CMs and Ed-DMD-iPSC-CMs. This result supported that impairment of actin stress fibers and decreased YAP activity deteriorated the proliferative ability of cardiomyocytes in DMD.

LPA promotes actin dynamics via the Rho-Rock kinase and increases YAP activity^22^. LPA stimulates cardiac differentiation and proliferation, and protects from myocardial infarction^35,36^. Our data showed that low concentrations of LPA improved YAP activity in DMD-iPSC-CMs. This might suggest that LPA treatment constitute cardiac protection in DMD cardiomyopathy. However, both low and high concentrations of LPA did not improve YAP activity in Con-iPSC-CMs and Ed-DMD-iPSC-CMs; high concentration of LPA had no additional effects in DMD-iPSC-CMs. We speculate that actin dynamics was at the highest in Con-iPSC-CMs and Ed-DMD-iPSC-CMs under these culture conditions; while it was stabilized by low concentration of LPA in DMD-iPSC-CMs. In addition, some studies have shown that LPA induces cardiac dysfunction^37^, increases CTGF-dependent fibroblast proliferation^38^, and stimulates myocardin-related transcription factor (MRTF)-A and MRTF-B, which activate profibrotic genes^39^. Thus, we should consider the side effects of LPA-induced fibrosis, as it might cause cardiac dysfunction.

Cardiac regeneration involves dedifferentiation, proliferation, and redifferentiation (DPR) following cardiac impairment^40,41^. The dedifferentiation and redifferentiation of cardiac iPSC-CMs are characterized by the de-organization of sarcomeric structures followed by the reorganization of these structures and restoration of cell morphology. Proliferation occurs in the early stages, between the dedifferentiation and redifferentiation. In this study, we confirmed the cardiac proliferative capacity a few days after seeding, following trypsinization. This represents the DPR process of iPSC-CMs, following cardiac damage. The altered YAP activity in DMD may be associated with the cardiac proliferation stage in the DPR process; however, the DPR process is not associated with DMD cardiomyopathy pathogenesis. The DPR process in DMD cardiomyopathy needs further elucidation.

In this study, there are several limitations to suggest altered YAP activity causes dystrophic heart as pathogenesis of DMD cardiomyopathy. Specifically, use of only a single cell line of DMD-iPSC-CMs does not fully reflect the in vivo phenotype and is not sufficient to conclusively determine the effect of YAP on dystrophic heart. We confirmed the proliferation ability and the actin dynamics of iPSC-CMs in the short term following seeding, but not in the long term. Therefore, it is unclear whether the regeneration process associated with YAP activity influences the progression of DMD cardiomyopathy. In addition, we must consider that the gene edited isogenic model of DMD-iPSCs has short length dystrophin without exon 48–55. Dystrophin with specific mutation of *DMD* gene is not adequate to elucidate the function of full-length dystrophin in association with YAP activity and actin dynamics. More evaluations in various mutation of *DMD* gene are currently required to research function of dystrophin with actin binding site.

In conclusion, we observed improved YAP activity and actin dynamics in DMD-iPSC-CMs following repair of dystrophin via CRISPR/Cas9. These results indicate that decreased YAP activity and impaired actin dynamics may contribute to the pathogenesis of DMD cardiomyopathy.

## Methods

### Clinical diagnosis

The patient was a male at 31 years old with muscle weakness and heart failure. The cardiac function was measured by echocardiography, which showed the left ventricular ejection fraction (LVEF) was 44%. He was diagnosed as DMD by the lack of dystrophin by staining of biopsied skeletal muscle. Genetic test, multiplex ligation-dependent probe amplification (MLPA) carried the deletion of exon 48–54 of *DMD* gene in this patient.

### iPSC culture

iPSCs were cultured on a dish coated with iMatrix-511 (Nippi) in StemFit AK02 (Ajinomoto). To maintain pluripotency, these iPSCs were enzymatically passaged with StemFit AK02 containing 10 µmol/L Y-27632 (Wako) at 80% confluence. iPSCs were maintained in StemFit AK02 without Y-27632 from the next day until the next passage. These iPSCs were cultured in the presence of 5% CO_2_ at 37 °C.

### Genotyping

Genomic DNA was extracted from iPSCs using the QIAmp DNA micro kit (Qiagen) to confirm the exon deletion. Polymerase chain reaction (PCR) was performed using KOD FX NEO (Toyobo) with primers designed on each exon. The amplified DNA was separated using electrophoresis on 2% agarose gel in Tris-borate-EDTA (TBE) buffer. The sequences of the primers used are shown in Supplemental Table 1.

### Editing of DMD-iPSCs using CRISPR/Cas9

We designed each single guide RNA (sgRNA) sequence upstream and downstream of exon 55 to change “out of frame” to “in frame” via non-homologous end joining (NHEJ) in DMD-iPSCs. The pX459 vector (Addgene, plasmid #48139)^42^ encoding these sgRNAs were amplified in DH5α competent cells. Two sgRNAs were co-transfected into DMD-iPSCs via electroporation (NEPA21 type II, NepaGene). Co-transfected DMD-iPSCs were purified by puromycin selection and single colony picking. The sgRNA sequences are shown in Supplemental Table 1.

### Sanger sequencing

Total RNA was extracted from iPSCs using the RNeasy Plus mini kit (Qiagen) and cDNA was synthesized using Super Script VILO (Thermo Fisher Scientific). PCR was performed using KOD FX NEO, with forward primer on exon 46 and reverse primer on exon 56. The amplified cDNA was separated via electrophoresis on 2% agarose gel in TBE buffer and evaluated using Sanger sequencing analysis at the Center for Medical Research and Education, Osaka University Graduate School of Medicine. The sequences of the forward and reverse primers are shown in Supplemental Table 1.

### Cardiomyocyte differentiation

The PSC cardiomyocyte differentiation kit (Thermo Fisher Scientific) was used to efficiently generate iPSC-CMs with some modifications. Undifferentiated iPSCs were seeded on a dish coated with 1% Geltrex LDEV-free reduced growth factor (Thermo Fisher Scientific). At 80% confluence, the culture medium was changed to StemFit AK02 containing 1% reduced Matrigel growth factor (Corning). The next day, the culture medium was changed to medium A containing 1% reduced Matrigel growth factor, and medium B and medium C of the PSC cardiomyocyte differentiation kit were added consecutively after every 2 days. Subsequently, these iPSCs was maintained in medium C.

### Preparation of iPSC-CMs

The iPSC-CMs were dissociated to single cells via trypsin-EDTA (0.25%) (Thermo Fisher Scientific) treatment for 10 min at 37 °C. After centrifugation at 160*g* for 5 min, the iPSCs were resuspended in Medium 199 (Thermo Fisher Scientific) containing 10% fetal bovine serum (FBS). These iPSCs were seeded on a 96-well imaging plate (PerkinElmer Cell Carrier) and glass bottom dish (Matsunami) coated with 1% Geltrex LDEV-free reduced growth factor. CytoSoft Imaging 24-well plate (ADM) with a thin layer of biocompatible silicone was used in the experiments as the hard and soft substrate.

### Immunofluorescent staining

iPSC-CMs on the imaging plate were fixed using 4% paraformaldehyde in phosphate buffered saline (PBS)(−), permeabilized using 0.1% Triton-X in PBS(−) for 15 min at 4 °C, and blocked with 5% bovine serum albumin (BSA) in PBS(−) for 60 min at room temperature. The samples were treated with primary antibodies of optimum dilutions for 24 h at 4 °C. Next, the samples were incubated with secondary antibodies of optimum dilution for 1 h at room temperature and the nuclei were labeled with Hoechst 33342 (1:1000, Dojindo). The primary and secondary antibodies used are shown in Supplemental Table 2.

### Actin staining

F and G actin were labeled using Alexa Fluor 568 phalloidin (Thermo Fisher Scientific, A12380) and Alexa Fluor 488 conjugated deoxyribonuclease I (Thermo Fisher Scientific, D12371), respectively.

### Imaging analysis using high content imaging system

Images of immunofluorescent staining were analyzed using the Operetta high content imaging system (PerkinElmer). N/C ratio, F/G actin ratio, and cell morphology were automatically detected by the Harmony analysis software (PerkinElmer). The imaging analysis was performed in 59 fields; the size of one field was 500 µm × 650 µm, and a number of cells were evaluated. We screened troponin T-positive cells in iPSC-CMs. YAP localization, dystrophin expression, Ki67 expression, cell morphology, and actin filament status were analyzed in troponin T-positive cells.

### Live cell imaging for visualizing actin dynamics

pCAG–LifeAct–TagRFP (Ibidi) was transfected in iPSC-CMs using Lipofectamine 3000 (Thermo Fisher Scientific). To label iPSC-CMs, adeno-associated virus (AAV) (Takara) encoding pCMV-EGFP-TNNT2 was constructed and transfected. After 2 days of co-transfection, the iPSC-CMs were seeded on glass bottom dish according to the preparation protocol of iPSC-CMs. Actin dynamics Actin dynamics was assessed in EGFP-positive cell. Live imaging of actin was performed using a LSM700 confocal laser-scanning microscope (Zeiss) in the presence of 5% CO_2_ at 37 °C. The time-lapse interval was 1 min and the Z-stack slices were 1 µm thick. Live cell imaging was analyzed using Imaris (Oxford Instruments).

### Western blotting

The total protein of iPSC-CMs was extracted using radioimmunoprecipitation assay (RIPA) buffer containing a phosphatase inhibitor and protease inhibitor for 30 min on ice. After centrifugation at 20,000*g* for 20 min, the supernatant was used as total protein lysate. The samples were separated via electrophoresis on 5–20% Extra PAGE One precast gel (Nacalai Tesque). The fractionated samples were transferred to a nitrocellulose membrane. The membrane was blocked using 5% BSA in Tris-buffered saline with Tween 20 (TBS-T) for 1 h at room temperature, followed by reaction with primary antibodies for 24 h at 4 °C. Next, the membranes were reacted with secondary antibody for 1 h at room temperature, followed by treatment with Pierce ECL Plus western blotting substrate (Thermo Fisher Scientific) for 5 min. The chemiluminescent signals were detected using the ECL Plus western blotting detection system (GE Healthcare Life Sciences) and the images were captured using ImageQuant LAS4000 (GE Healthcare Life Sciences). The primary and secondary antibodies used are shown in Supplemental Table 3.

### Statistical analysis

All data are presented as mean ± standard deviation (SD). Wilcoxon test was used for comparison between two groups; Steel–Dwass test, for among ≥ 3 groups, as non-parametric tests. Student’s *t *test was used for analysis between two groups; one-way analysis of variance (ANOVA) with Tukey–Kramer test, for comparison among ≥ 3 groups, as parametric tests. Parametric tests were applied after confirmation of normality using Levene test. *P* < 0.05 was considered statistically significant. N.S. indicates not significant. All statistical analyses were performed using JMP Pro 15.2 (SAS).

### Ethical approval

Written informed consent was obtained from the donor or their guardians under the protocol approved by the Institutional Review Board of Osaka University (Approval number 13254 (829-1)-3) and The University of Tokyo (G10019). This study conforms to the principles outlined in the Declaration of Helsinki.

### Disclosures

J-LEE: Joint Research Grant (SCREEN Holdings Co. Ltd, Alpha MED Scientific, Inc.).

## Supplementary Information

Supplementary Video 1.

Supplementary Video 2.

Supplementary Video 3.

Supplementary Video 4.

Supplementary Video 5.

Supplementary Video 6.

Supplementary Video 7.

Supplementary Video 8.

Supplementary Information 1.
