# Supplementary material for: Decreased YAP activity reduces proliferative ability in human induced pluripotent stem cell of duchenne muscular dystrophy derived cardiomyocytes
Source: Sci Rep. 2021 May 14;11:10351. doi: 10.1038/s41598-021-89603-8 (PMC8121946; doi:10.1038/s41598-021-89603-8)
Supplement: Supplementary file 9 — Supplementary Information 1. [file 41598_2021_89603_MOESM9_ESM.pdf]

Supplementary Information

Decreased YAP Activity Reduces Proliferative Ability in Human Induced Pluripotent Stem Cell of Duchenne Muscular Dystrophy Derived Cardiomyocytes

Hideki Yasutake[1 2], Jong-Kook Lee[2*], Akihito Hashimoto[1 3], Kiyoshi Masuyama[1], Jun Li[1 2], Yuki Kuramoto[1], Shuichiro Higo[4], Shungo Hikoso[1], Kyoko Hidaka[5], Atsuhiko T. Naito[6], Shigeru Miyagawa[7], Yoshiki Sawa[7], Issei Komuro[8], Yasushi Sakata[1]

[1]Department of Cardiovascular Medicine, Osaka University Graduate School of Medicine, 2-2 Yamadaoka, Suita 565-0871, Japan
[2]Department of Cardiovascular Regenerative Medicine, Osaka University Graduate School of Medicine, 2-2 Yamadaoka, Suita 565-0871, Japan
[3]Daihatsu Health Care Center, 2-1-1 Momozono, Ikeda 563-0045, Japan
[4]Department of Medical Therapeutics for Heart Failure, Osaka University Graduate School of Medicine, 2-2 Yamadaoka, Suita, Osaka 565-0871, Japan
[5]Center for Fundamental Education, The University of Kitakyushu, 4-2-1 Kokura Minami-ku Kitagata, Kitakyushu 802-8577, Japan
[6]Department of Physiology, Faculty of Medicine, Toho University, 5-21-16 Omori-nishi, Ohta-ku, Tokyo 143-8540, Japan
[7]Department of Cardiovascular Surgery, Osaka University Graduate School of Medicine, 2-2 Yamadaoka, Suita 565-0871, Japan
[8]Department of Cardiovascular Medicine, Graduate School of Medicine, The University of Tokyo, 7-3-1 Hongo, Bunkyo-ku, Tokyo 113-8655, Japan

***Correspondence:** Jong-Kook Lee, Department of Cardiovascular Regenerative Medicine, Osaka University Graduate School of Medicine, 2-2 Yamadaoka, Suita 565-0871, Japan, E-mail jlee@cardiology.med.osaka-u.ac.jp

Supplemental Figure

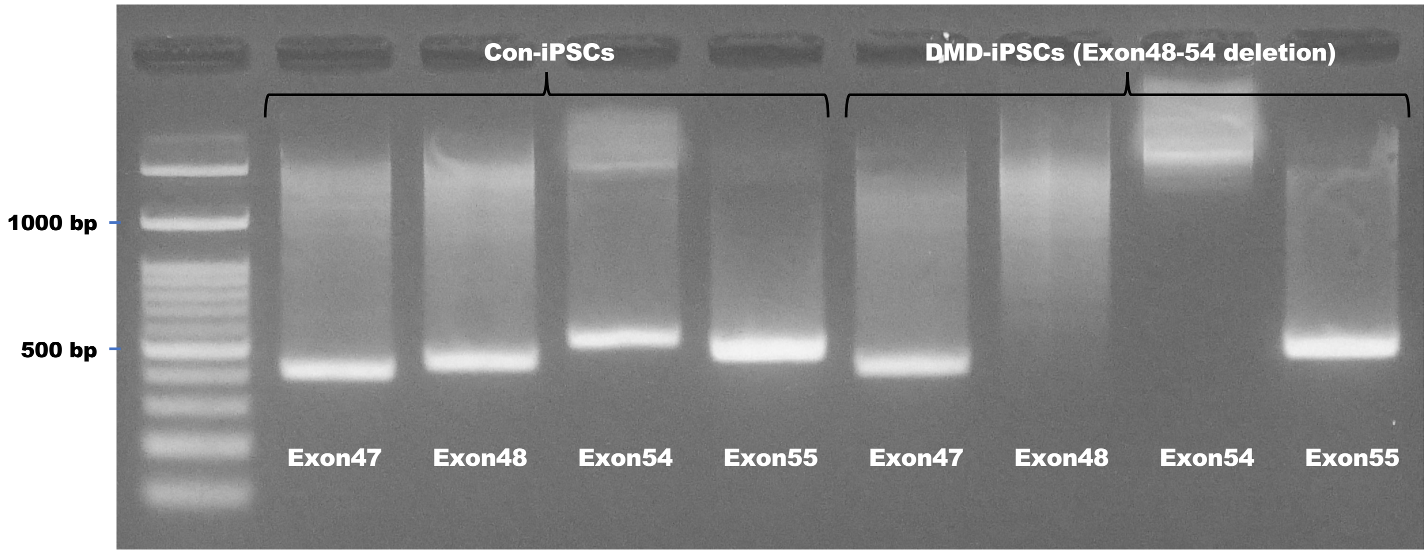

Supplemental Figure I. Genotyping of Con-iPSCs and DMD-iPSCs using PCR for each exon in DMD.

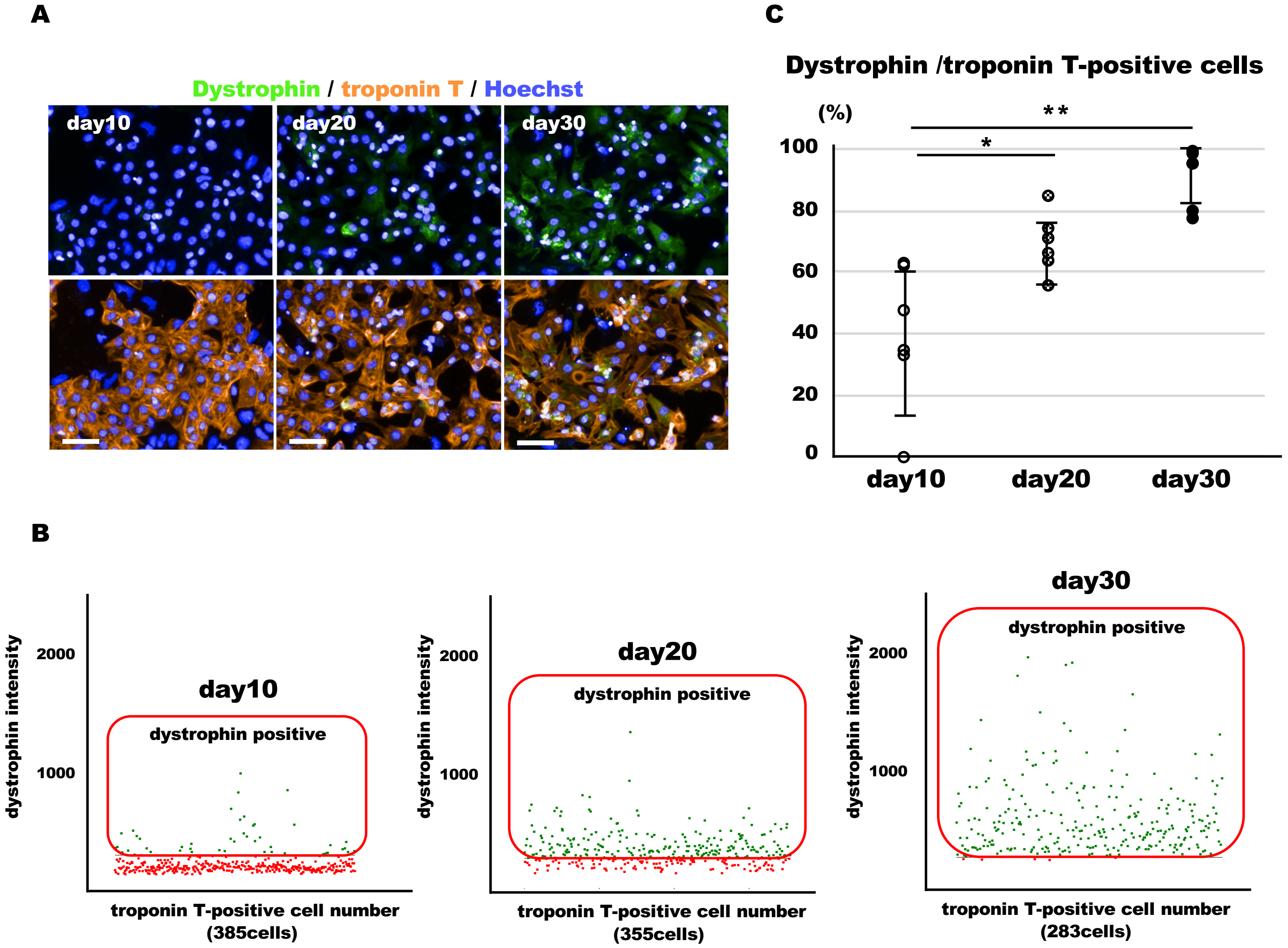

Supplemental Figure II. **A,** Dystrophin expression in Con-iPSC-CMs according to time course of cardiac differentiation demonstrated using immunofluorescence staining (Scale bar: 50 μm). **B,** Scatter plots of one field in Con-iPSC-CMs according to the time course of cardiac differentiation. Red frames indicate dystrophin-positive cells in troponin T-positive cells. **C,** Percentage of dystrophin-positive cells in troponin T-positive cells was evaluated in Con-iPSC-CMs. (n = 6 sessions, mean analyzed troponin T-positive cell number = 3202 ± 1739 cells, mean ± SD; *$P < 0.05$, **$P < 0.01$).

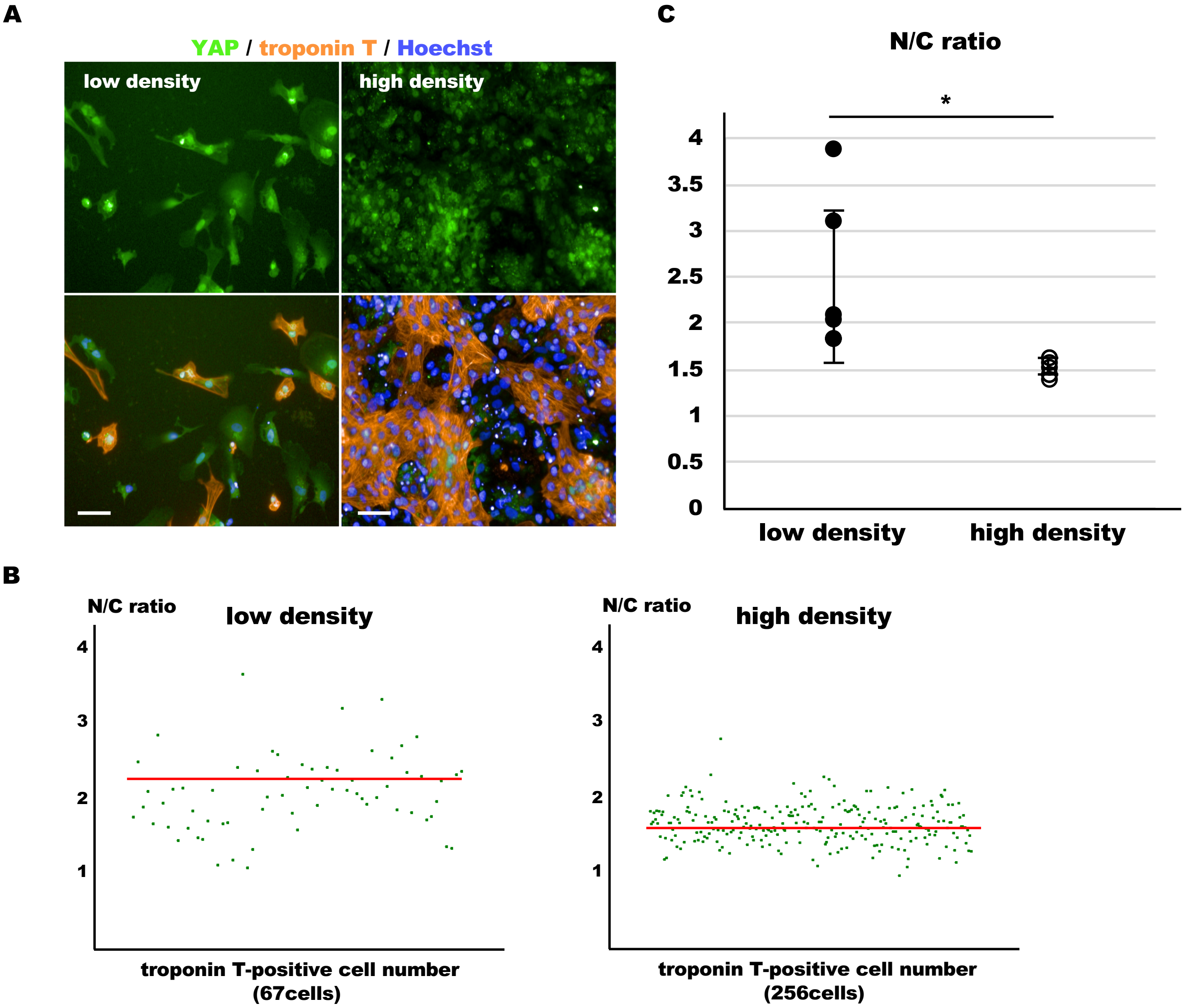

Supplemental Figure III. A, YAP localization was demonstrated using immunofluorescence staining in low and high cell densities (Scale bar: 50 μm). **B,** Scatter plots of one field in low density and high density in Con-iPSC-CMs. Red bars indicate mean N/C ratio in troponin T-positive cells. **C,** N/C ratio in troponin T-positive cells was evaluated in low and high cell density (n = 7 sessions, mean analyzed troponin T-positive cell number at low density: 403 ± 228 cells, at high density: 6906 ± 1285 cells, mean ± SD; **P* < 0.05).

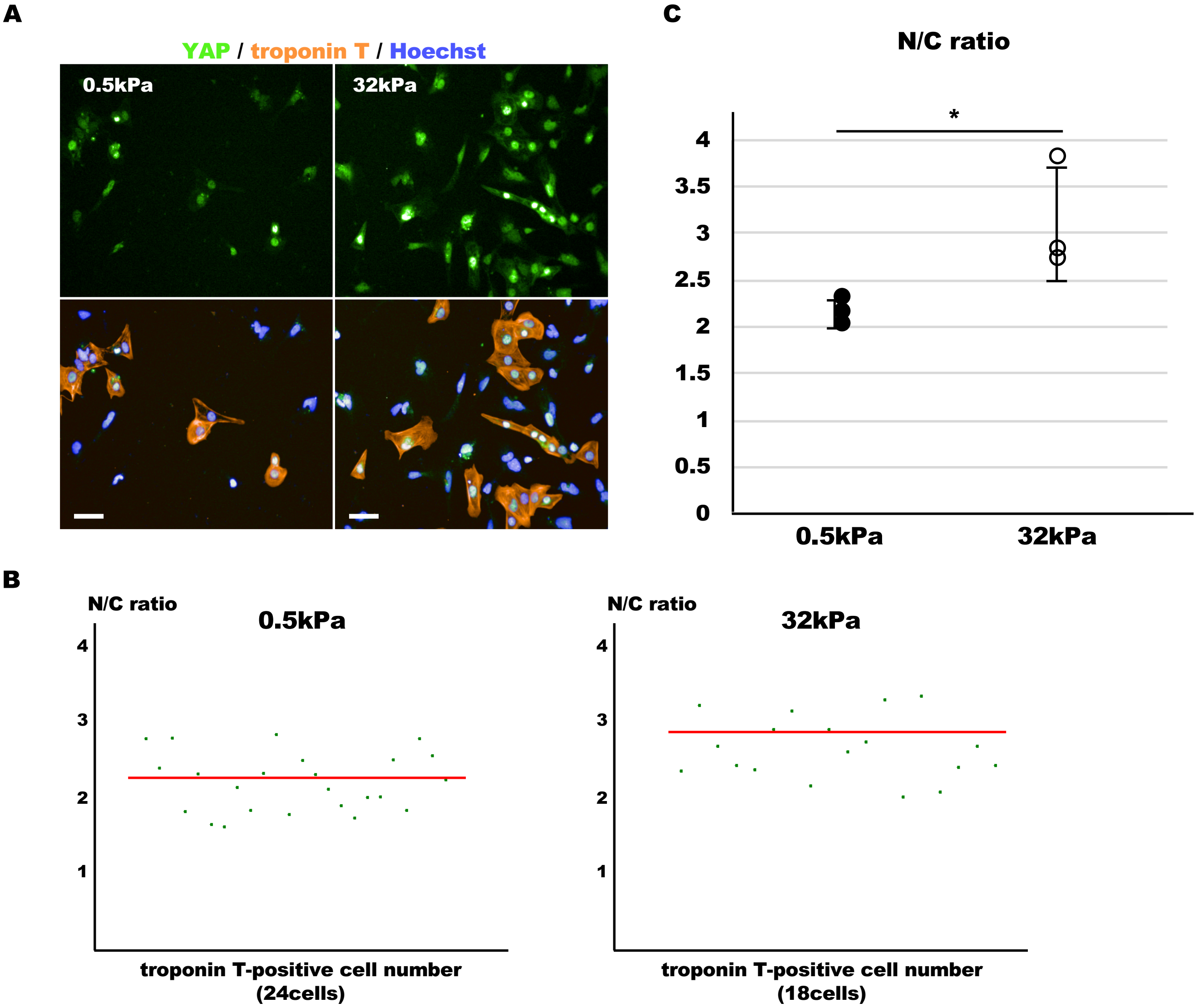

Supplemental Figure IV. A, YAP localization was demonstrated using immunofluorescence staining in soft substrate (0.5 kPa) and hard substrate (32 kPa) (Scale bar: 50 µm). **B,** Scatter plots of one field in soft substrate (0.5 kPa) and hard substrate (32 kPa) in Con-iPSC-CMs. Red bars indicate mean N/C ratio in troponin T-positive cells. **C,** N/C ratio in troponin T-positive cells was evaluated in soft substrate (0.5 kPa) and hard substrate (32 kPa) (n = 3 sessions, mean analyzed troponin T-positive cell number at 0.5 kPa: 1535 ± 664 cells, at 32 kPa: 1649 ± 1493 cells, mean ± SD, **P* < 0.05).

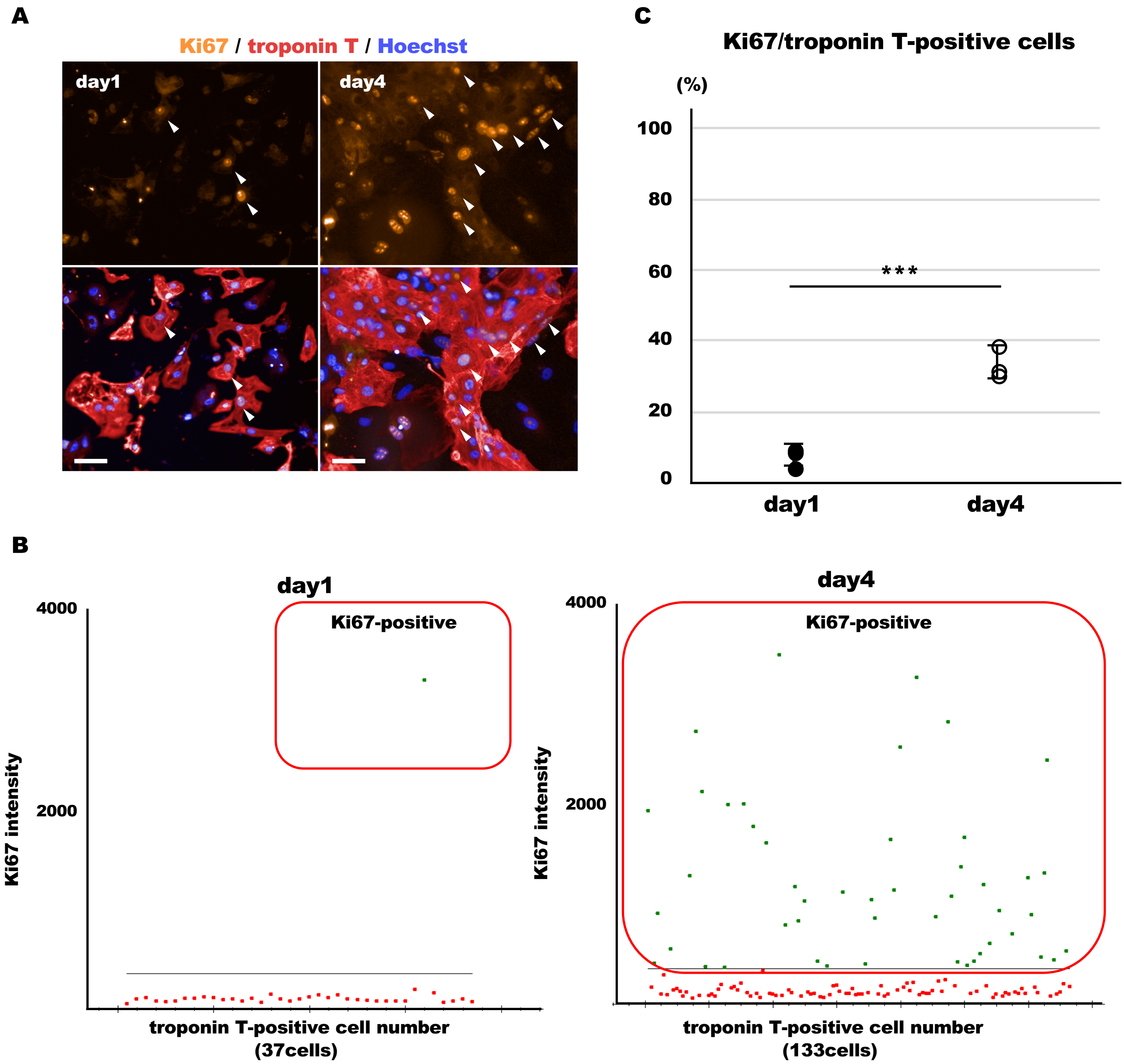

Supplemental Figure V. A, Ki67 expression was demonstrated using immunofluorescence staining in Con-iPSC-CMs, day1 and day4 from seeding. Arrowheads indicate Ki67-positive cells in troponin T-positive cells (Scale bar: 50 μm). **B,** Scatter plots of one field in Con-iPSC-CMs according to the time course from seeding. Red frames indicate Ki67-positive cells in troponin T-positive cells. **C,** Percentage of Ki67-positive cells in troponin T-positive cells was evaluated in Con-iPSC-CMs day1 and day4 from seeding (n = 3 sessions, mean analyzed troponin T-positive cell number on day 1: 1060 ± 274 cells, on day 4: 3972 ± 2129 cells, mean ± SD, ***$P < 0.001$).

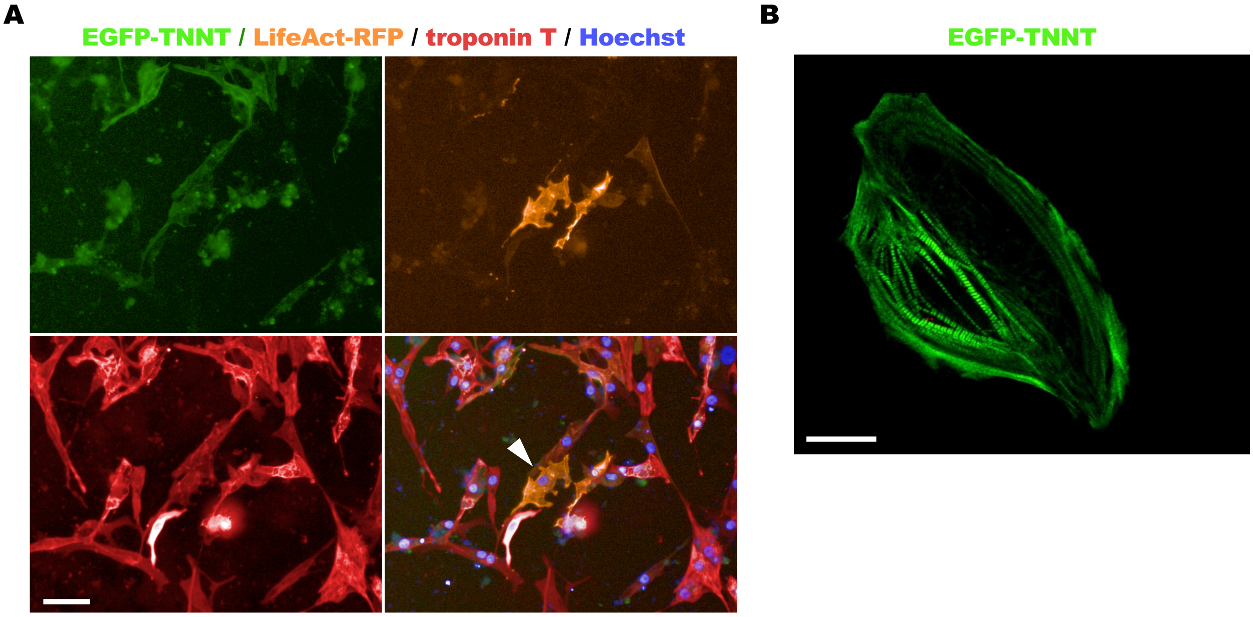

Supplemental Figure VI. A, Immunofluorescence image using troponin T of iPSC-CMs co-transfected with EGFP-TNNT and LifeAct-RFP (Scale bar: 50 µm). Arrowheads indicate both EGFP and RFP expression in troponin T-positive cells. **B,** EGFP-TNNT revealed a sarcomere structure in iPSC-CMs (Scale bar: 20 µm).

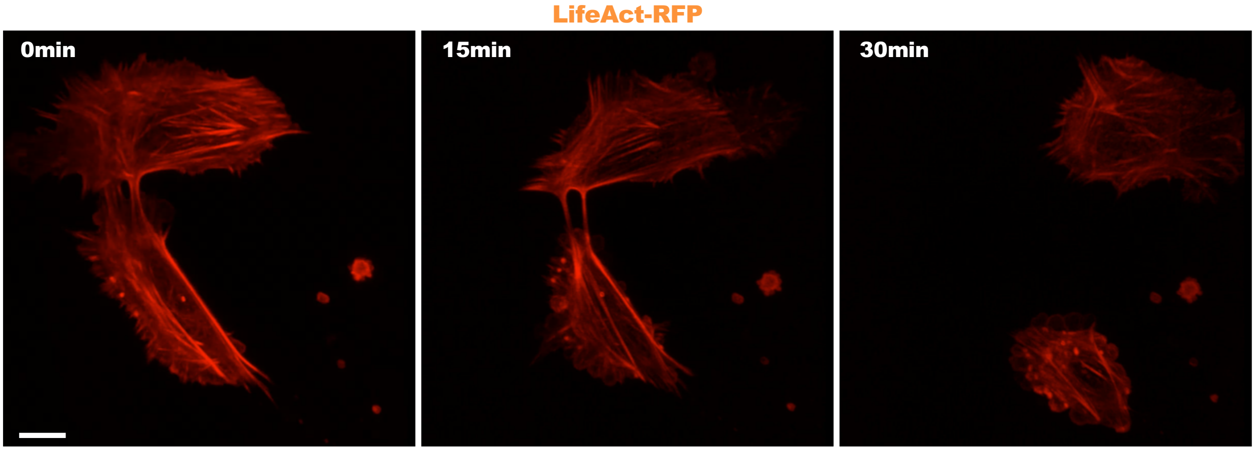

Supplemental Figure VII. The images demonstrate non-cardiomyocyte of iPSCs during cell division with actin dynamics (Scale bar: 10 µm) (Movie 1).

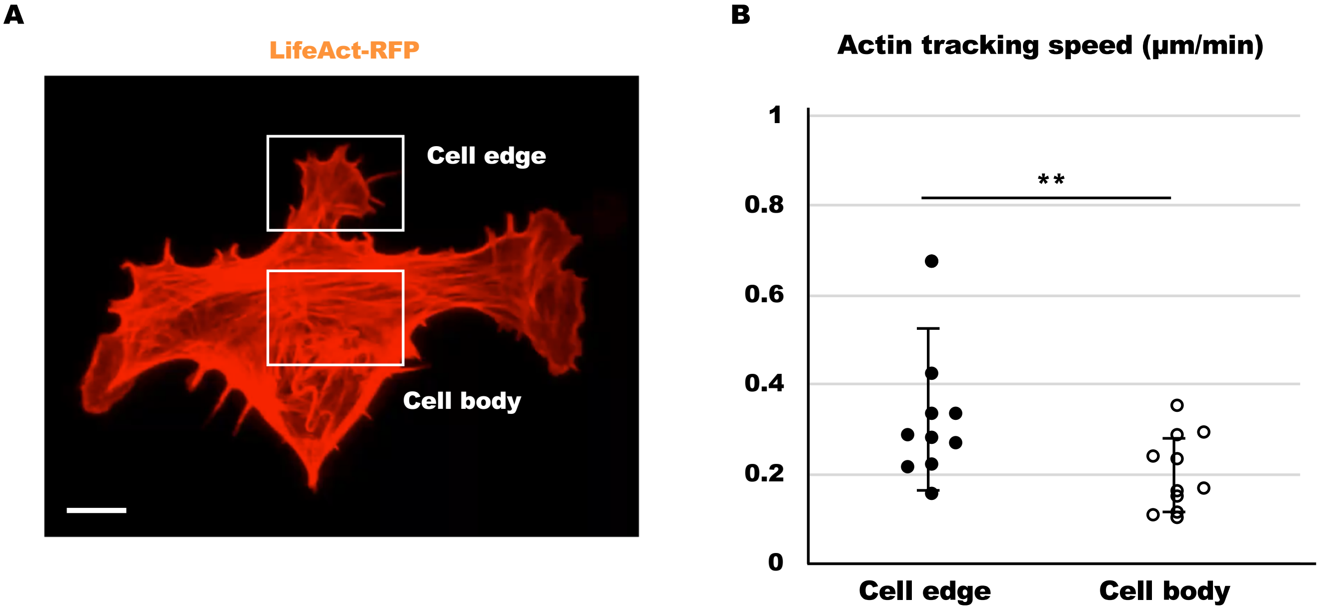

Supplemental Figure VIII. A, Actin dynamics in Con-iPSC-CMs was demonstrated using live cell imaging (Scale bar: 10 μm) (Movie 2). White squares frames show cell edge and cell body. **B,** Actin tracking speed was estimated at the cell edge and cell body (n = 4 sessions, 11 cells, mean ± SD, $^{**}P < 0.01$).

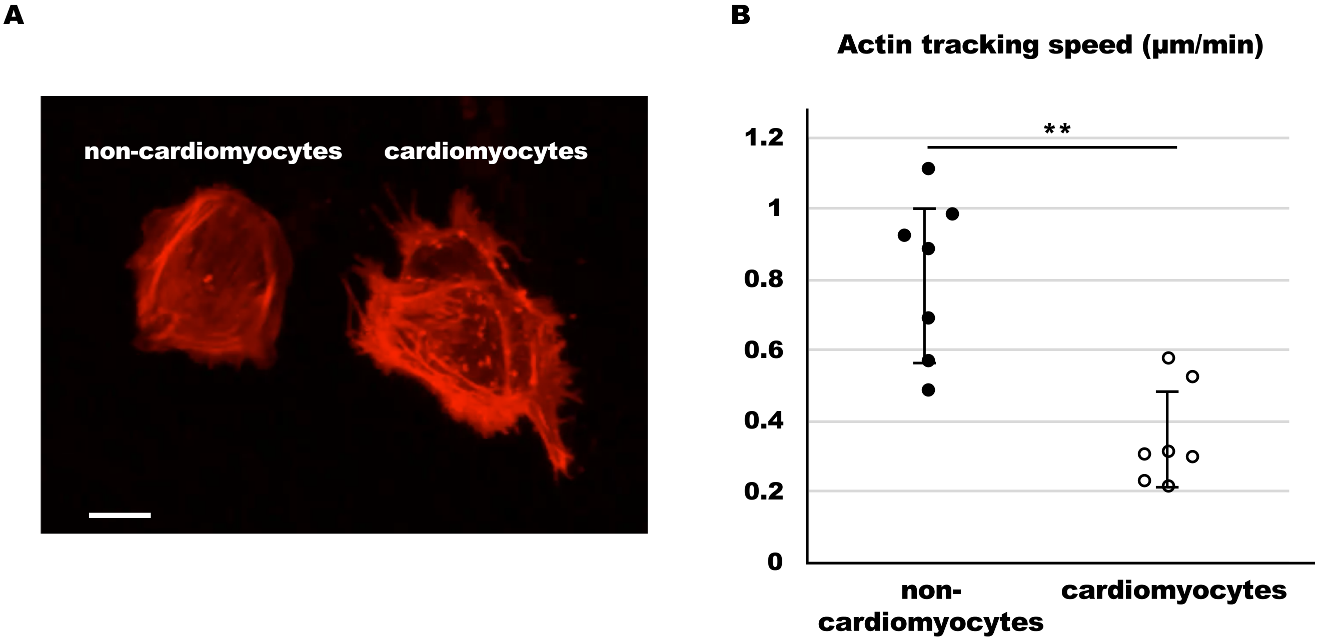

Supplemental Figure IX. A, The images demonstrate actin dynamics in non-cardiomyocytes and cardiomyocytes of Con-iPSCs (Scale bar: 10 µm) (Movie 3). **B,** Actin tracking speed was estimated at the cell edge of non-cardiomyocytes and cardiomyocytes (n = 3 sessions, 7 cells, mean ± SD, **P < 0.01).

Supplemental Table

Target of sgRNA	Sequence
Upstream of exon55	caccGCAACAACTCACCCCATTGT
Upstream of exon55-anti	aaacACAATGGGGTGAGTTGTTGC
Downstream of exon55	caccGTACTTGTAACTGACAAGCC
Downstream of exon55-anti	aaacGGCTTGTCAGTTACAAGTAC

Target of DMD exon	Sequence
Exon 47 Forward	GTCAATCAGCTCTGTGCTCA
Exon 47 Reverse	ACAACAATCCACATACCAGCCT
Exon 48 Forward	GCCTTTGTGTAAGGTGTGTG
Exon 48 Reverse	CGTCAAATGGTCCTTCTTGG
Exon 54 Forward	TCCTGAAAGGTGGGTTACCT
Exon 54 Reverse	GTCTGAGCCAAGTCCGTGAGT
Exon 55 Forward	CCCCATACAAACGCCTTTAAG
Exon 55 Reverse	GTTTTGTCCCTGGCTTGTCAGT
Exon 46-56 Forward	GGAGGAAGCAGATAACATTGCT
Exon 46-56 Reverse	ACGTCTTTGTAACAGGACTGC
Exon 47-56 Forward	AGTGCTCCCATAAGCCCAGAAG
Exon 47-56 Reverse	GCATCATCGGAACCTTCCAGG

Supplemental Table 1. Designed sgRNA sequences for DMD-iPSCs and primers of genotyping for DMD-iPSCs

**Name**	**Maker, Catalog number**	**dilution**
Anti-Dystrophin	Abcam, ab15277	1:300
Anti-Cardiac Troponin T	Abcam, ab8295	1:300
Anti-Cardiac Troponin T	Abcam, ab45932	1:300
Anti-Cardiac Troponin T	Abcam, ab64623	1:300
Anti-YAP1	Santa Cruz Biotechnology, sc101199	1:200
Ki67	Abcam, ab15580	1:500

**Name**	**Maker, Catalog number**	**dilution**
Alexa Fluor 488 donkey anti-mouse IgG (H+L)	Thermo Fisher Scientific , A21202	1:300
Alexa Fluor 568 donkey anti-rabbit IgG (H+L)	Thermo Fisher Scientific , A10042	1:300
Alexa Fluor 647 donkey anti-goat IgG (H+L)	Life technologies, A21447	1:300

Supplemental Table 2. Immunofluorescence primary antibodies and second antibodies.

Name	Maker, Catalog number	dilution
Anti-Dystrophin	Abcam, ab15277	1:500
Anti-YAP1	Santa Cruz Biotechnology, sc101199	1:200
GAPDH	CST, 14C10	1:10000

Name	Maker, Catalog number	dilution
Peroxidase AffiniPure Donkey Anti-Rabbit IgG (H+L)	Jackson, 711-035-152	1:5000

Supplemental Table 3. WB primary antibodies and second antibodies
